# Biochemical and pharmacological prospects of *Citrus sinensis* peel

**DOI:** 10.1016/j.heliyon.2022.e09979

**Published:** 2022-07-21

**Authors:** Doha H. Abou Baker, Bassant M.M. Ibrahim, Yasmin Abdel-Latif, Nabila S. Hassan, Emad M. Hassan, Souad El Gengaihi

**Affiliations:** a Medicinal and Aromatic Plants Research Department, Pharmaceutical and Drug Industries Research Institute, National Research Centre, Dokki, Giza, PO 12622, Egypt; b Pharmacology Department, Medicine and Clinical Studies Research Institute, National Research Centre, Dokki, Giza, PO 12622, Egypt; c Faculty of Biotechnology, October University for Modern Sciences and Arts, 6th October, Giza, Egypt; d Pathology Department, Medical Research Institute, National Research Centre, Dokki, Giza, PO 12622, Egypt

**Keywords:** Gastric ulcer, Citrus peels, Hesperidin, Hepatoprotective, Antioxidant, Anti-inflammatory, Gastroprotective

## Abstract

Gastric ulcer and hepatotoxicity due to irrational drug overuse are two of the most serious conditions associated with inflammation and oxidative stress that affect the digestive system. This study aimed to experimentally evaluate the hepatoprotective/gastroprotective effects of aqueous and butanol citrus peel extracts and hesperidin in rat models of ulcer and hepatotoxicity. Acute toxicity study was performed for determining the safe dose of citrus extracts to analyze efficacy. In the experiments on hepatoprotective and gastroprotective effects, rats were classified into nine groups in each experiment: (1) negative control, (2) positive control hepatotoxic model with paracetamol (640 mg/kg)/gastric ulcer model:ethanol 70% (1 ml), (3)reference hepatoprotective:silymarin (25 mg/kg)/gastroprotective:ranitidine (50 mg/kg), and (4–9) groups treated for 2 weeks before induction of each disease with either citrus aqueous or butanol extracts or hesperidin (125–250 mg/kg). Drugs, ethanol, or tested compounds were administered orally. The levels of biochemical parameters, such as AST, ALT, NO, MDA, CRP, and ILβ6, were significantly reduced, but CAT level was increased. Postmortem examination of liver and stomach tissues of treated animals revealed marked improvement compared with positive control animals. Hesperidin exerted the best hepatoprotective, antioxidant, anti-inflammatory, and gastroprotective effects, followed by butanol and then aqueous citrus peel extracts.

## 1 Introduction

Inflammation is body’s defensive mechanism against injury caused by traumatic triggers (Ibrahim et al., 2016a). Inflammatory triggers stimulate innate immunity in cases of severe tissue damage, wherein there is an increased production of proinflammatory cytokines and chemokines (Mostafa et al., 2016).

Among two of the most serious digestive system diseases, gastric ulcer occurs due to excessive ethanol intake, overuse of nonsteroidal anti-inflammatory drugs, smoking, and oxidative stress (Ibrahim et al., 2016b). The other disease is hepatotoxicity due to drug abuse, which is highly common because some drugs such as paracetamol are metabolized into more toxic compounds than the parent compound (Mori et al., 2003). Oxidative stress also causes hepatocyte insult that in turn may progress to hepatic neoplasms (Mansour et al., 2019).

Since several drugs used for the treatment of gastric ulcers such as the H_2_ antagonist “ranitidine” and current drugs used for the management of hepatic diseases exert undesirable side effects, it is necessary to develop newer, safe, natural anti-inflammatory agents (Ibrahim et al., 2016a). This can be achieved through herbal approach for the management of inflammatory diseases, which is considered to be beneficial for strengthening the body in disease condition (Taha et al., 2016). Herbal extracts can be used for the prevention of several nervous, cardiovascular, and digestive system diseases and other systemic diseases depending on their anti-inflammatory and antioxidant abilities, which are in turn due to their rich contents of polyphenols (coumarins, tannins, and flavonoids) (El-Gengaihi et al., 2016a, 2016b; 2020; Ibrahim et al., 2016a, 2016b, 2016c; Mossa et al., 2015; Salam et al., 2016, Shaaban et al., 2016; Allam et al., 2018; Abou Baker, 2020a, 2020b; Abou Baker and Rady, 2020; Abou Baker et al., 2020a, 2020b).

In the past four decades of the twentieth century, there has been the maximum production of citrus fruits. Orange constitutes the largest single production of citrus fruits and currently contributes to >60% of the total world production. Approximately two-thirds of citrus fruits produced worldwide is consumed as fresh fruit.

Citrus has also been the source of the distinctive flavor that has been appreciated by people throughout the world for centuries (Rouseff and Perez-Cacho, 2007).

Botanically, citrus is a part of the family Rutaceae, subfamily Aurantioideae, containing the following six closely related genera: *Citrus*, *Fortunella*, *Poncitrus*, *Microcitrus*, *Eremocitrus*, and *Clymentia*. Most flavors of commercial values are found in the genus *Citrus* and the subgenus *Eucitrus*.

Sweet orange variety is the major fruit produced worldwide. The major cultivars of commercial importance include Valencia, Pera, Navel, Hamlin, and Fellers (1985).

In Egypt, the entire orange cultivation area is 374,559 feddan, with a total productivity of 2,855,022 metric tons, including all types of orange.

Valencia and Navel orange are the two important types. A large amount of byproducts are generated after the processing of citrus fruits, which contain valuable compounds (El-gengaihi et al., 2020).

Citrus waste contains soluble sugars, starch, cellulose and hemicellulose fibers, lignin, pectin, and other bioactive compounds. The presence of these bioactive compounds renders citrus waste harmful to the environment. Citrus peel and pulp are the major byproducts of juice-processing industries, representing approximately 55%–60% of fresh fruit weight. The waste from processing industries was estimated to be 15 × 10^6^ tons worldwide (Kalra et al., 1989).

The phytochemicals from citrus waste are used in cosmetic formulations for hair and skin, such as antifungal soaps, and in several other compounds that help in curing obesity. Different medical effects of citrus-derived phytochemicals have been reported, for example, effects against type 2 diabetes (Johnston et al., 2005) and cancer (Edwards-Jones et al., 2004).

This study was conducted to determine the protective effects of citrus butanol and aqueous extracts and hesperidin isolated from *Citrus sinensis* peels on some digestive system inflammatory conditions such as gastric ulcer and hepatotoxicity. These effects were evaluated by investigating the antioxidant and anti-inflammatory activities of the extracts and hesperidin.

## 2 Material and methods

### 2.1 Plant material

Peels removed from mature Navel orange were cut into small pieces then allowed to dry in oven at 50 °C. The dried peel was powdered then kept in paper pages till used.

### 2.2 Extraction

The dried citrus peel of navel orange was extracted with pet-ether in a soxhlet apparatus. The pet. ether was discarded, then the dried powdered as extracted by acetone at room temperature. The acetone was evaporated, and the residue obtained was partitioned with hexane, chloroform, ethyl acetate, butanol and finally with water. Each extract was concentrated and each residue was kept in a brown bottles till used.

#### 2.2.1 Isolation of hesperidin from dried orange peels

The dried orange peels was placed in an extraction sleeve of a soxhlet apparatus using pet-ether (40–60°), then the pet-ether was discarded. The powdered peel is laid out to remove the pet. ether then again put in the extraction sleeve and extracted with methanol until colorless (1–2 h). The methanolic extract was filtered and the filtrate was acidified by 6% of acetic acid (pH 3–4). The content liquid is kept under cooling (4–6 °C) over night until a solid crystalline substance appears. It was then filtered and the crude hesperidin was separated out as amorphous substance, upon crystallization it yields yellowish brown needles (47.75%). Its purity was checked by thin layer chromatography. Crude hesperidin gave red color with ferric chloride test whereas it gave violet color on Shinoda test. Two spots were observed in thin layer chromatography of crude hesperidin using n-Butanol: Acetic Acid: Water (3:1:1) as mobile phase at 0.20 and 0.62 Rf according to published literature (Abou Baker et al., 2020a, 2020b). The flavonoid glycoside, hesperidin, colourless needles were separated and used for the investigation.

#### 2.2.2 Chemical investigation

HPLC analysis of the butanol and water extracts of orange peel was performed using the following condition.

Liquid chromatogram equipped with an auto sampler and diode array detector. The analytical column was an eclipse XDB-C18 (150 × 4.6 μm; 5 μm) with C18 guard column. The injection vol. was 50 μl and peaks were monitored simultaneously at 280 and 320 nm for the benzoic and cinnamic acid derivatives respectively. All samples were filtered through a 0.45 μm Acrodisc sgringe filter (Gelman laboratory MI) before injection. Peaks were identified by congruent retention times and uv spectra and compared with those of authentic.

#### 2.2.3 Determination of total phenolics and antioxidant activity of the peels

The total phenolic content of the butanol and aqueous extracts were analyzed using Folin–Ciocâlteu reagent according to the method of Singleton and Rossi (1965) using gallic acid as standard while free radical scavenging capacity (DPPH & ABTS) for both extracts were determined adopted the method of Hwang and Do Thi (2014).

### 2.3 Pharmacological effects

#### 2.3.1 Materials and Methods

**Ι. Animals:**

Wistar male albino rats, weighing ranged from 150 to 175 g were used for acute toxicity study. In addition to other animals of the same weight were used for determination of the hepato-protective effects, anti-oxidant activities and anti-ulcerative activities of citrus aqueous and butanol extracts and hesperidin. The animals were obtained from the animal house colony of the National research centre, Dokki, Giza, Egypt. The animals were housed in standard metal cages in an air conditioned room at 22 ± 3 °C, 55 ± 5% humidity and provided with standard laboratory diet and water *ad libitum*. All experimental procedures were conducted in accordance with the guide for care and use of laboratory animals with approval number 16/138 obtained from Ethics Committee of the National Research Centre and followed the recommendations of the National Institutes of Health Guide for Care and Use of Laboratory Animals (Publication No. 85-23, revised 1985).

**II. Drugs:**

a) Ranitidine obtained from Boehringer Ingelheim GmbH, b)Paracetamol (acetaminophen) powder obtained from EIPICO, Egypt, c) Silymarin powder obtained from SEDICO, pharmaceutical Co., 6 October City-Egypt.

**III. Chemicals:**

- Ethanol (MERCK Co. Inc-Rahaway, NJ, USA)
- Diethyl ether, Formaldehyde (Sigma Chemical Co., St. Louis, MO, USA)

**IV. Diagnostic kits:**

- Kits for determination of ALT (alanine aminotransferase), AST (aspartate aminotransferase) in serum, and kits for determination of Lipid peroxides content (MDA), Nitric oxide content (NO) and Catalase content (CAT) in liver tissue homogenates (Biodiagnostic company, Egypt).
- Elisa Kits for determination of C reactive protein (CRP) and Interleukin beta 6(ILβ6) in serum.

#### 2.3.2 Experimental methods

In vivo biological studies were conducted to determine some pharmacological activities of citrus aqueous extract, hesperidin and citrus butanol extract:

##### 2.3.2.1 Acute toxicity study

Healthy young adult male Wister albino rats weighing from 150 to 175 g were used in the experiment. The animals were kept for five days before the test under housing and feeding conditions mentioned before. Animals were kept fasting overnight, then weighed and the doses of aqueous and butanol extracts that would be given to each rat were calculated according to body weight, extracts were prepared just before administration orally to five male rats in each group in doses of 2.5 g/kg dissolved in 2 mL distilled water. Another five male rats served as negative controls and were given 2 mL of distilled water. Animals were observed individually once during the first 30 min after dosing, then periodically during the first 24 h with special attention during the first 4 h. After which the animals were observed for changes in behaviour, bowel habits, obvious weight loss and mortality during the next 14 days following administration of the extracts. Acute toxicity study was done according to OECD test guideline 425 (2008).

There weren’t any mortalities or toxicity signs detected, during the duration of 14 days, hence acute toxicity study revealed that aqueous and butanol extracts were non-toxic in doses up to 2.5 g/kg b.w. So the experimental doses used in the present prophylactic efficacy study were 1/20 and 1/10 of (2.5 g/kg) of citrus butanol and aqueous extracts (125 and 250 mg/kg).

##### 2.3.2.2 Prophylactic efficacy study

**1. Hepatoprotective studies of citrus aqueous, butanol extract, and hesperidin**

**1.2. Experimental Design**

Seventy two rats were divided into nine groups each of eight animals as following:

Group (1) Negative control group: Rats received daily oral dose of 1 ml distilled water, served as negative control.

Group (2) Positive control group: Rats received paracetamol orally in a dose of 640 mg/kg (Moharram et al., 2018).

Treated groups:

Group (3) Reference group: Rats received silymarin orally (25 mg/kg) (Bhandari et al., 2003) daily for two successive weeks before paracetamol (640 mg/kg) was given orally.

Groups (4&5): Rats received citrus aqueous extract orally (125 and 250 mg/kg) . Groups (6&7) received hesperidinorally (125 and 250 mg/kg) (Abou Baker et al., 2020a, 2020b).Groups (8&9) received citrus butanol citrus extract orally (125 and 250 mg/rat)

One hour after the last doses of either silymarin or citrus aqueous, butanol extracts and hesperidin administration, paracetamol (640 mg/kg) was given orally.

**2. Evaluation of the hepato-protective effects of citrus aqueous, butanol extracts or** hesperidin

**2.1. Biochemical parameters**

At the end of the experimental period (24 h after paracetamol injection), the blood was obtained from all groups of rats after being lightly anaesthetized with ether by puncturing retro-orbital plexus Sorg and Buckner, 1964, the blood was allowed to flow into a clean dry centrifuge tube and left to stand 30 min before centrifugation to avoid hemolysis. Then blood samples were centrifuged for 15 min at 2500, rpm the clear supernatant serum was separated and collected by Pasteur pipette into a dry clean tube to use for determination of serum levels U/L of: Alanine aminotransferase (ALT) and Aspartate aminotransferase (AST) according to the method of Reitman and Frankel (1957).

**2.2. Histopathological study**

Animals were sacrificed 24 h after the last treatment, the thoracic cavities opened, livers rapidly and carefully excised and all attached vessels and ligaments trimmed off. The removed livers were washed with cold saline, dried with filter papers and weighed, then dropped into a jar containing 10% formalin as a fixative and kept for histo-pathological examination Liver slides were prepared and stained with hematoxylin and eosin (H& E) staining (Bancroft and Gamble, 2008).

**3. Study of anti-oxidant activities citrus aqueous extract, citrus butanol extract and hesperidin in liver and stomach tissues**

**3.1. Preparation of tissue homogenate**

Animals were sacrificed 24 h after the last treatment, the thoracic and abdominal cavities opened, livers and stomachs were rapidly and carefully excised and all attached vessels and ligaments were trimmed off. Tissues were kept in −80° freezers till homogenization. From every animal in each group one part of the liver or the stomach tissue was added to 4 parts of the ice cold normal saline (0.9%) and homogenized using a homogenizer then the homogenates were centrifuged at 4000 rpm for 15 min using a cooling centrifuge at 4 °C. The supernatant was removed and used in estimation of biochemical parameters (Hussein et al., 2012).

**3.2. Evaluation of antioxidant activity**

3.2.1. Determination of lipid peroxides content (MDA nmol/g.tissue)in liver or stomach homogenates according to method of Ruiz-Larrea et al. (1994).
3.2.2. Determination of catalyse content (CAT μ/g.tissue)in liver or stomach homogenates according to method of Johansson and Borg (1988)
3.2.3. Determination of Nitric oxide content (NO μmol/g.tissue) in liver or stomach homogenates according to method of Miranda et al. (2001)

**4. Study of gastro-protective effect of citrus aqueous extract, hesperidin and citrus butanol extract**

**4.1. Experimental design**

Seventy two rats were divided into nine groups each of eight animals as following:

Group (1) Negative control group: Rats received daily oral dose of 1 ml distilled water, served as negative control.

Group (2) Positive control group: Rats received ethanol 70% orally in a single dose of 1 ml/rat (Alkofahi and Atta, 1999).

Treated groups:

Group (3) Reference group: Rats received ranitidine orally (50 mg/kg) (Alvarez et al., 1999), daily for two successive weeks before ethanol 70% (1 ml/rat) was given orally 1 h after the last dose.

Groups (4&5): Rats received citrus aqueous extract orally (125 and 250 mg/kg). Groups (6&7) received hesperidin orally (125 and 250 mg/kg) .Groups (8&9) received citrus butanol citrus extract orally (125 and 250 mg/rat)

All treated groups received treatments orally for two successive weeks. Oral administration of ethanol (70 % 1 ml/kg) was administered orally 1 h on day 14 after the last treatment dose to the 2nd to 9th groups.

**5. Evaluation of the anti-inflammatory and gastro-protective effects of citrus aqueous extract, hesperidin and citrus butanol extract**

**5.1. Biochemical parameters**

At the end of the experimental period (one hour after ethanol administration), the blood was obtained from all groups of rats for determination of serum levels of systemic inflammatory marker C-reactive protein (CRP) and interleukin beta six (ILβ6) according to the manufacturer kit using rat ELISA kits.

Then all animals were sacrificed by cervical dislocation and their stomachs were excised for macroscopic, microscopic examination and biochemical measurement of antioxidant activity (NO, CAT and MDA).

**5.2. Macroscopic examination**

The stomachs of all rats in all groups were opened along the greater curvatures and gently rinsed with 0.9% NaCl. Gross mucosal lesions were recognized as hemorrhage or erosions with damage to the mucosal surface. The number and severity of mucosal lesions were noted and lesions were scored as follows:0: No lesion, 0.5: Diffuse hyperemia, 1: 1 to 2 small ulcers, 1.5: 3 to 6 small ulcers, 2: 7 to 10 small ulcers, 2.5. More than 10 small ulcers, 3: 1 marked ulcer plus 0 to 4 small ulcers, 3.5: 1 marked ulcer plus 5 or more small ulcers, 4: 2 marked ulcers plus 0 to 4 small ulcers, 4.5: 2 marked ulcers plus 5 or more small ulcers, 5: 3 or more marked ulcers (Clementi et al., 1998).

**5.3. Histopathological study**

Immediately after macroscopic evaluation the stomachs were fixed in neutral buffered 10% normal saline for 72 h at least. All the specimens were washed in tap water for half an hour and then dehydrated in ascending grades of alcohol (70%–80%–90% and finally absolute alcohol), cleared in xylene, impregnated in soft paraffin wax at 55 °C and embedded in hard paraffin. Serial sections of 6 μm thick were cut and stained with Haematoxylin and Eosin (Drury and Wallington, 1980) for histopathological investigation.

### 2.4 Statistical analysis

The data are expressed as mean ± SE for each group. Results of biochemical tests were analyzed using one-way analysis of variance, followed by the Tukey–Kramer test for multiple comparisons; P value of less than 0.05 was considered significant in all types of statistical tests. Graph Pad Software (Graph Pad Software Inc., La Jolla, CA, USA) (version 7) was used to carry out the statistical analysis tests.

## 3 Results

### 3.1 Chemical investigation

Table 1 shows the data of total phenolics and the antioxidant activity of butanol and aqueous extracts. The total phenol contents were almost similarat22.780 and 23.188 μg GAE/mg in the butanol and aqueous extracts, respectively.

**Table 1:** Total phenolic and antioxidant capacity of extracts.

	Total phenol μg GAE/mg	DPPH mg/Trolox/g	ABTS mg/Trolox/g
Aqueous	23.188 ± 0.41	9.182 ± 0.15	37.26 ± 0.33
Butanol extract	22.780 ± 0.09	7.971 ± 0.19	37.63 ± 0.28

(GAE-gallic acid equivalent).

The antioxidant activities were 7.971 and 9.182 mg/Trolox, as evaluated using the DPPH method, but 37.63 and 37.26 mg/Trolox, as evaluated using the ABTS method, for the two extracts, respectively.

When analyzing these two extracts by HPLC the data reported in Table 2 represents its constituents.

**Table 2:** HPLC analysis of the phenolic compounds in the two extracts.

Compounds	Conc.(μg/g)	
	Butanolic extract	Aqueous extract
Gallic acid	79298.7	19243.5
Chlorogenic acid	5522.0	2706.9
Catechin	ND	2814.7
Methyl gallate	ND	5.4
Caffeic acid	1159.9	6.8
Syringic acid	327.1	381.5
Pyro catechol	881.6	ND
Rutin	105.7	35.2
Ellagic acid	10124.7	23.5
Coumaric acid	464.7	3.3
Vanillin	22.4	ND
Ferulic acid	567.9	627.4
Naringenin	1000.1	621.4
Hesperidin	1526.7	2086.7
Taxifolin	103.7	5.5
Cinnamic acid	6.0	ND
Kaempferol	11.0	ND

### 3.2 Pharmacological studies

#### 3.2.1 Acute toxicity study

Based on the results of the acute toxicity study, the selected experimental doses were 1/20 and 1/10 of 2.5 g/kg of citrus aqueous and butanol extracts (125 and 250 mg/kg), respectively. The dose of hesperidin was also 125 and 250 mg/kg, respectively (Abou Baker et al., 2020a, 2020b).

#### 3.2.2 Prophylactic efficacy study of citrus aqueous and butanol extracts and hesperidin (125 and 250 mg/kg)

##### 3.2.2.1 Hepatoprotective effect

The citrus aqueous and butanol extracts and hesperidin exerted significant lowering effects on hepatic enzyme levels compared with the hepatotoxic group, as high doses of all the tested agents exerted better effects than low doses; however, these effects were not dose-dependent. The best effects were exerted by hesperidin and butanol extract administer red at high doses, wherein the AST levels were 31.93 and 28.75 U/L, respectively, and of the ALT levels were 20.68 and 21.23 U/L, respectively (Table 3).

**Table 3:** Hepatoprotective effects of the two citrus extracts and hesperidin.

Group	Parameter	
	AST (U/L)	ALT (U/L)
Negative Control	29.98 ± 1.18	18.15 ± 0.71
Positive control group Paracetamol (640 mg/kg)	91.4 ± 1.39^@^	63.77 ± 2.28^@^
Reference group Silymarin (25 mg/kg)	33.25 ± 0.43∗	21.5 ± 1.27∗
Aqueous citrus extract (125 mg/kg)	48.8 ± 1.17^@^∗^$^	30.78 ± 1.94^@^∗^$^
Aqueous citrus extract (250 mg/kg)	39.55 ± 1.76 ^@^∗^&^	24.28 ± 1.49∗
Butanol citrus extract (125 mg/kg)	35.42 ± 1.87 ∗^&^	37.78 ± 1.7 ^@^∗^$ˆ#^
Butanol citrus extract (250 mg/kg)	28.75 ± 1.47∗^&ˆ!£^	???
Hesperidin (125 mg/kg)	40.23 ± 0.43 ^@^∗^&^	31.63 ± 0.78^@^∗^$^
Hesperidin (250 mg/kg)	31.93 ± 1.55∗^ˆ&!^	20.68 ± 1.28∗^&!^

Results are expressed as mean of levels ± S.E of AST and ALT in serum of rats treated with aqueous citrus extract, hesperidin, and butanol citrus extract (125 and 250 mg/kg) and silymarin (25 mg/kg) for two successive weeks followed by induction of hepatotoxicity by using paracetamol (640 mg/kg), N = 8, Data were analysed using one way analysis of variance (ANOVA) followed by Tukey Kramer multiple comparisons test; Significant at P ≤ 0.05.

@ Significant different from negative control group; ∗Significant difference from positive control group.

$ Significant difference from silymarin group; &Significant difference from the citrus aqueous extract (125 mg/kg) group; ˆSignificant difference from the citrus aqueous extract (250 mg/kg) group; £Significant difference from the butanol citrus extract(125 mg/kg) group; !Significant difference from the hesperidin (125 mg/kg) group; #Significant difference from the hesperidin (250 mg/kg) group.

##### 3.2.2.2 Antioxidant effects on experimentally induced hepatotoxicity and gastric ulceration in rats

Regarding the antioxidant effects in the hepatotoxic model, the two extracts and hesperidin exerted significant antioxidant effects, which were evident by the significant reduction of NO and MDA levels and the elevation of CAT levels compared to those in the hepatotoxic group wherein oxidative stress was induced using paracetamol (640 mg/kg). Moreover, high doses of all the tested agents exerted better effects than low doses, but these effects were not dose-dependent. The best effects were exerted by hesperidin and butanol extract administered at high doses, wherein the NO levels were 10.88 and 9.97 μmol/g, respectively, and the CAT levels were 5.2 and 3.47 μ/g, respectively. The best effects on MDA levels were exerted by aqueous and butanol extracts administered at high doses, at which the MDA levels were 65.3 and 59.68 nmol/g, respectively (Table 4).

**Table 4:** Antioxidant activity of the two citrus extracts and hesperidin in rat model of hepatotoxicity.

Group	Parameter		
	No (μmol/g Tissue)	CAT (μ/g Tissue)	MDA (nmol/g tissue)
Negative Control	7.95 ± 0.67	3.6 ± 0.23	34.05 ± 0.72
Positive Control Group Paracetamol (640 mg/kg)	34.1 ± 1.15^@^	1.33 ± 0.04^@^	167.3 ± 3.11^@^
Reference group Silymarin (25 mg/kg)	10.15 ± 0.67∗	2.7 ± 0.23∗	44.05 ± 0.72^@^∗
Aqueous citrus extract (125 mg/kg)	16.57 ± 0.5^@^∗^$^	2.56 ± 0.06∗	83.03 ± 2.61^@^∗^$^
Aqueous citrus extract (250 mg/kg)	14.15 ± 0.47^@^∗^$^	2.9 ± 0.3∗	65.3 ± 2.46^@^∗^$&^
Butanol citrus extract (125 mg/kg)	14.15 ± 0.47®∗^$#^	2.57 ± 0.08∗^#^	70.43 ± 1.89^@^∗^$&ˆ^
Butanol citrus extract (250 mg/kg)	9.97 ± 0.55∗^&£^	3.47 ± 0.17∗^#^	59.68 ± 1.37^@^∗^$&ˆ^
Hesperidin (125 mg/kg)	11.7 ± 0.24^@^∗^&^	3.2 ± 0.09∗^#^	95.4 ± 1.1^@^∗^$&ˆ^
Hesperidin (250 mg/kg)	10.88 ± 0.28∗^&^	5.2 ± 0.474^@^∗^$&ˆ^	66.73 ± 2.88^@^∗^$&ˆ^

Results are expressed as mean of levels + S.E of nitric oxide (NO), catalase (CAT) and malondialdehyde (MDA) in liver homogenates of rats treated with aqueous citrus extract, hesperidin, and butanol citrus extract (125 and 250 mg/kg) and silymarin (25 mg/kg) for two successive weeks followed by induction of hepatotoxicity by using paracetamol 640 mg/kg; n = 8; Data were analysed using one way analysis of variance (ANOVA) followed by Tukey Kramer multiple comparisons test; Significant at P < 0.05 @ Significant different from negative control; ∗Significant difference from positive control group; $ Significant difference from silymarin group; &Significant difference from the aqueous extract (125 mg/kg) group; A Significant difference from the aqueous extract (250 mg/kg) group; !Significant difference from the methanol extract (125 mg/kg) group; # Significant difference from the methanol extract (250 mg/kg) group; £ Significant difference from the butanol extract (125 mg/kg) group.

Regarding the gastric ulceration model also, all the three tested agents administered at both high and low doses exerted significant antioxidant effects, which were evident by the significant reduction of NO and MDA levels and elevation of CAT levels compared to those in the ulcer group wherein oxidative stress was induced using 70% ethanol (1 ml/rat). Furthermore, high doses of both aqueous extracts and hesperidin exerted better effects than low doses on NO levels (14.9 and 11.3 μmol/g, respectively) compared with the positive control group (46.8 μmol/g). Regarding their effects on CAT levels, high doses of aqueous and butanol extracts and hesperidin exerted better effects than low doses (2.85, 3.2, and 4.55 μ/g, respectively) compared with the positive control group (0.95 μ/g). For MDA levels, only the high dose of butanol extract exerted the best lowering effect (64.6 nmol/g) compared with the positive control group (173.7 nmol/g) (Table 5).

##### 3.2.2.3 Gastroprotective effect

All the three tested agents resulted in a significant decrease in the number of gastric ulcers in the macroscopic examination of stomachs extracted from groups treated before the administration of 70% ethanol (l ml/rat) compared with the untreated group that received only 70% ethanol (1 ml/rat) without prior treatment. The effects of low doses were better than those of high doses. The effects of both extracts and hesperidin were better than those of ranitidine (50 mg/kg). Butanol extract at low dose exhibited the best result as the number and severity of ulcers were 0.33 and 0.33 compared with the positive control (4 and 3.5) and those of ranitidine (1.16 and 0.66), respectively (Table 6).

##### 3.2.2.4 Anti-inflammatory activity against 70% ethanol

Low and high doses of the three agents exhibited significant anti-inflammatory activities manifested by the reduction of CRP and IL-β6 levels in the serum of groups treated before the administration of 70% ethanol (1 ml/rat) compared with the untreated group that received only 70% ethanol (1 ml/rat) without prior treatment. The effect of hesperidin at high dose was the best as the CRP level was 1.83 ng/ml compared with the positive control group (3.45 ng/ml) and ranitidine group (2.2 ng/ml), and the IL-β6 level was 41.67 pg/ml compared with the positive control group (103.8 pg/ml) and ranitidine group (47.3 pg/ml) (Table 7).

##### 3.2.2.5 Histopathological results

**Figure 1:**
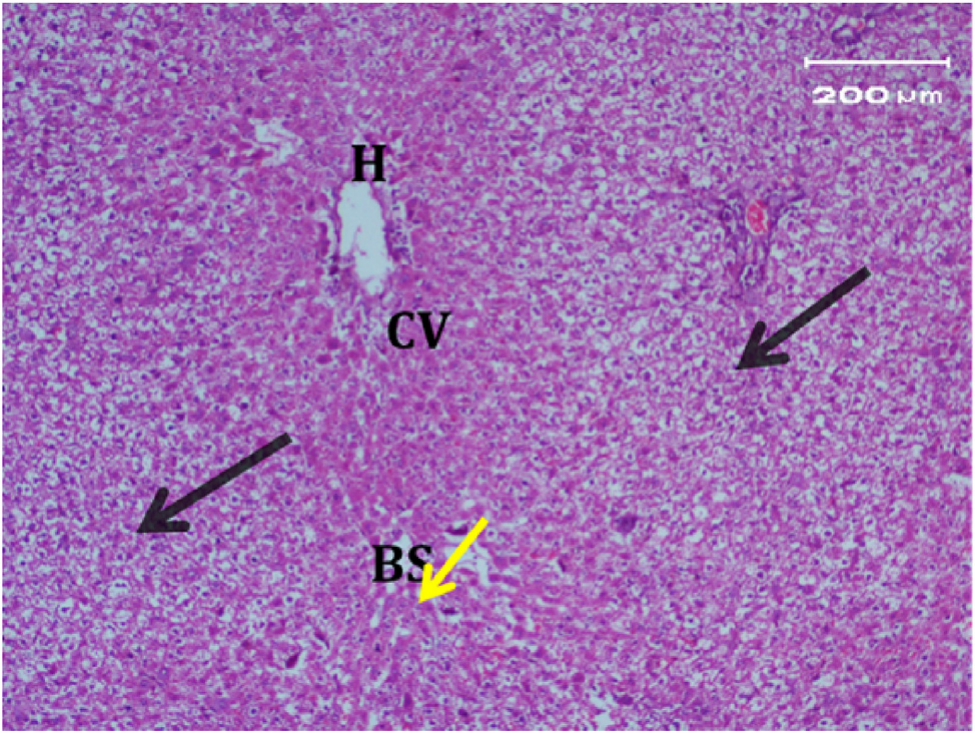
Light micrograph of a negative control rat liver (H&E. ×400).

**Figure 2:**
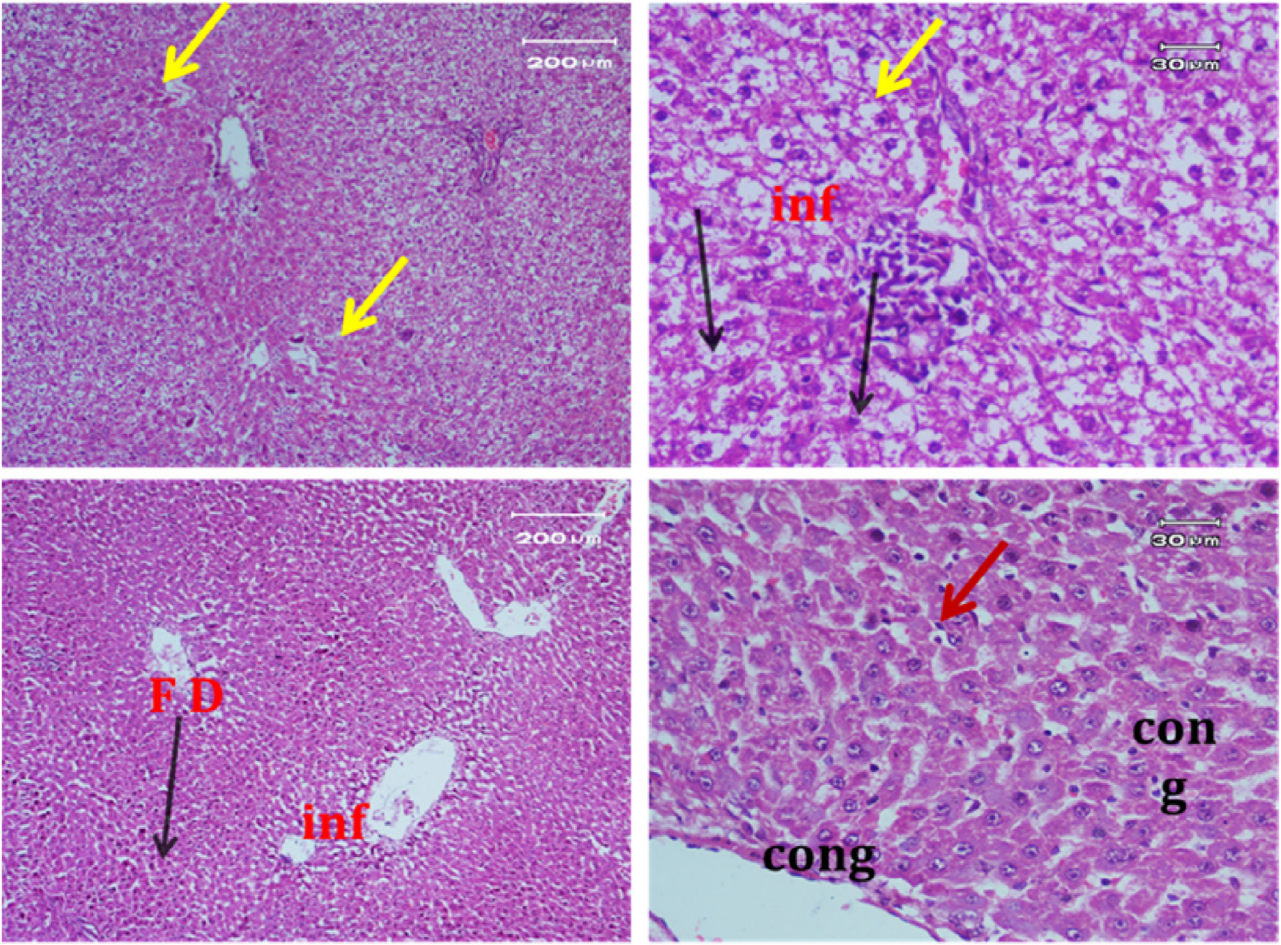
Light micrograph of a positive control rat liver tissue.

Light micrograph of a negative control rat liver tissue (Figure 1) revealed normal hepatocytes and cords radiating from the central vein with blood sinusoids in between (BS)with rounded vesicular nuclei and granular cytoplasm (black arrow). In contrast, the light micrograph of a positive control rat liver tissue under paracetamol (640 mg/kg) treatment only (Figure 2) revealed periportal necrosis, fibrosis, inflammatory cell infiltration, bands of connective tissues (inf), and hypertrophy of portal triad and bile duct (red arrow).

**Figure 3:**
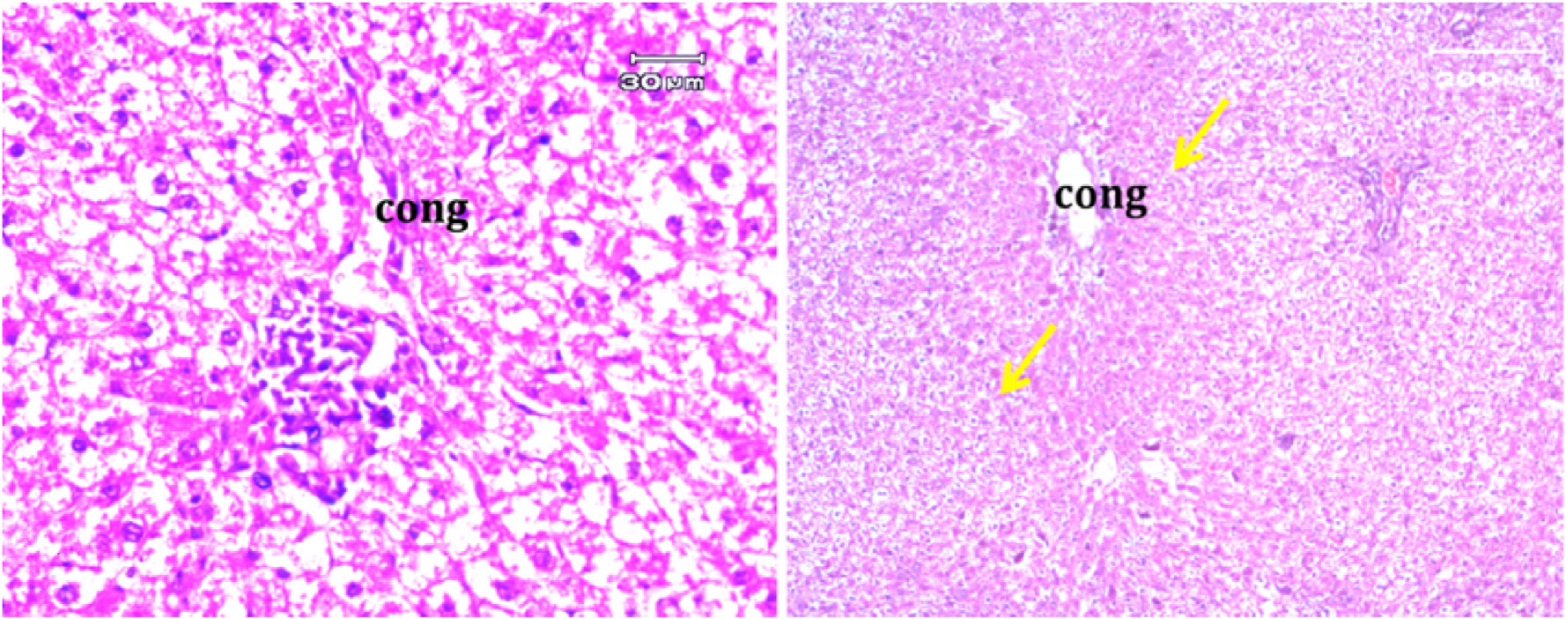
Light micrograph of a reference group rat liver tissue (H&E. 100 × 400).

**Figure 4:**
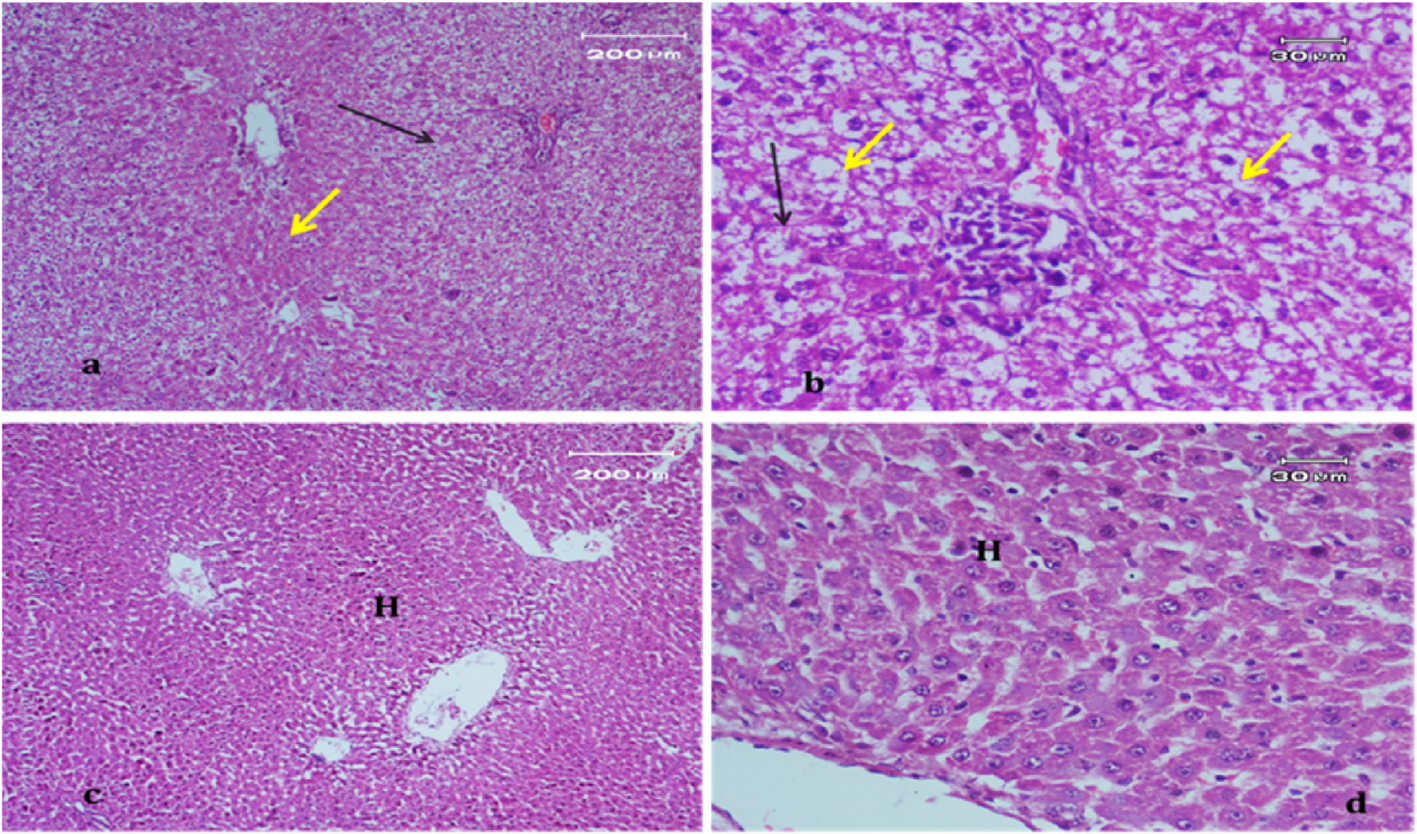
Light micrograph of a rat liver tissue treated with Low dose of aqueous citrus extract (a,b), and high dose (c&d) (H&E. 100 × 400).

**Figure 5:**
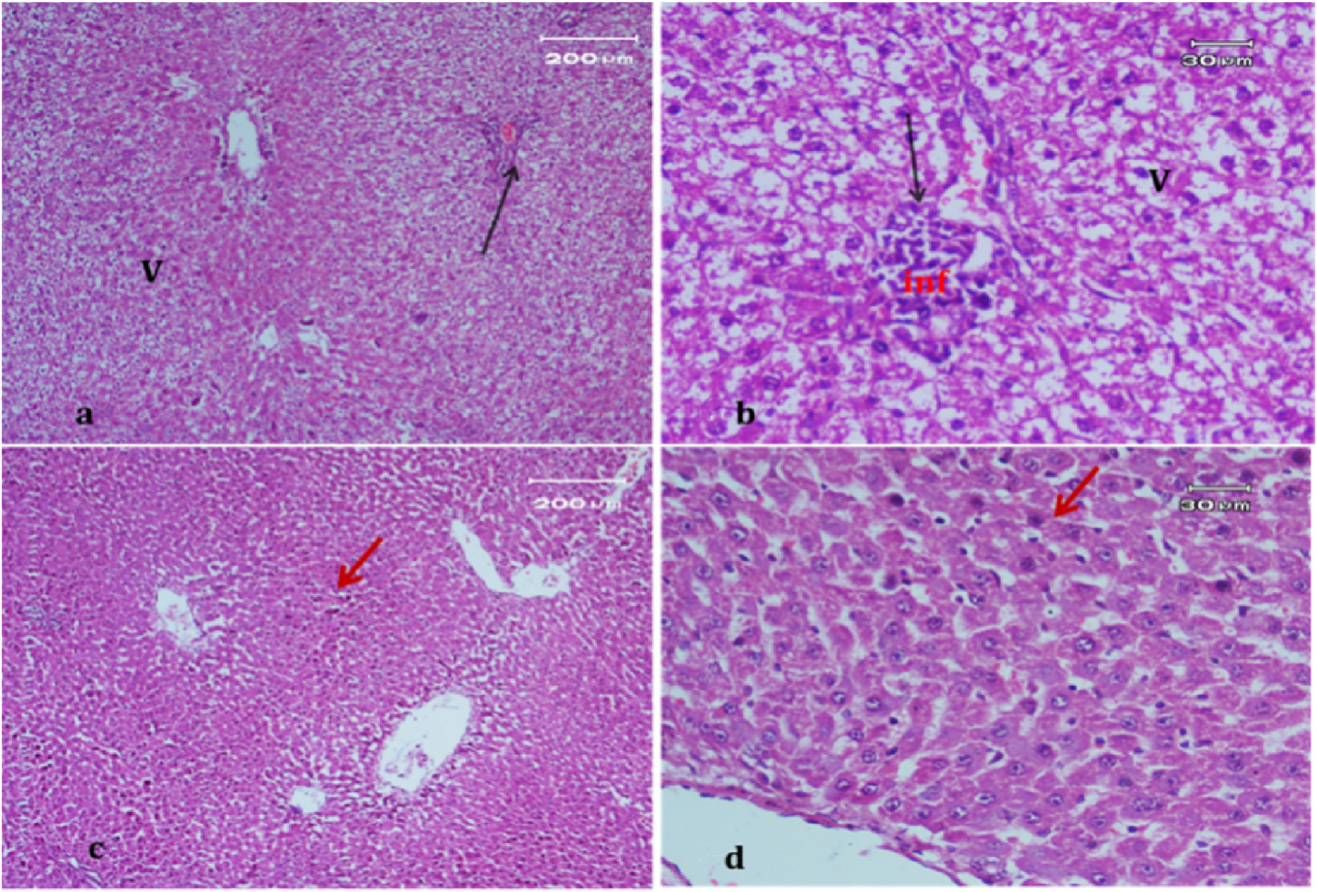
Light micrograph of a rat liver tissue treated with Low dose of hesperidin (a,b) and high dose (c&d) (H&E. 100 × 400).

**Figure 6:**
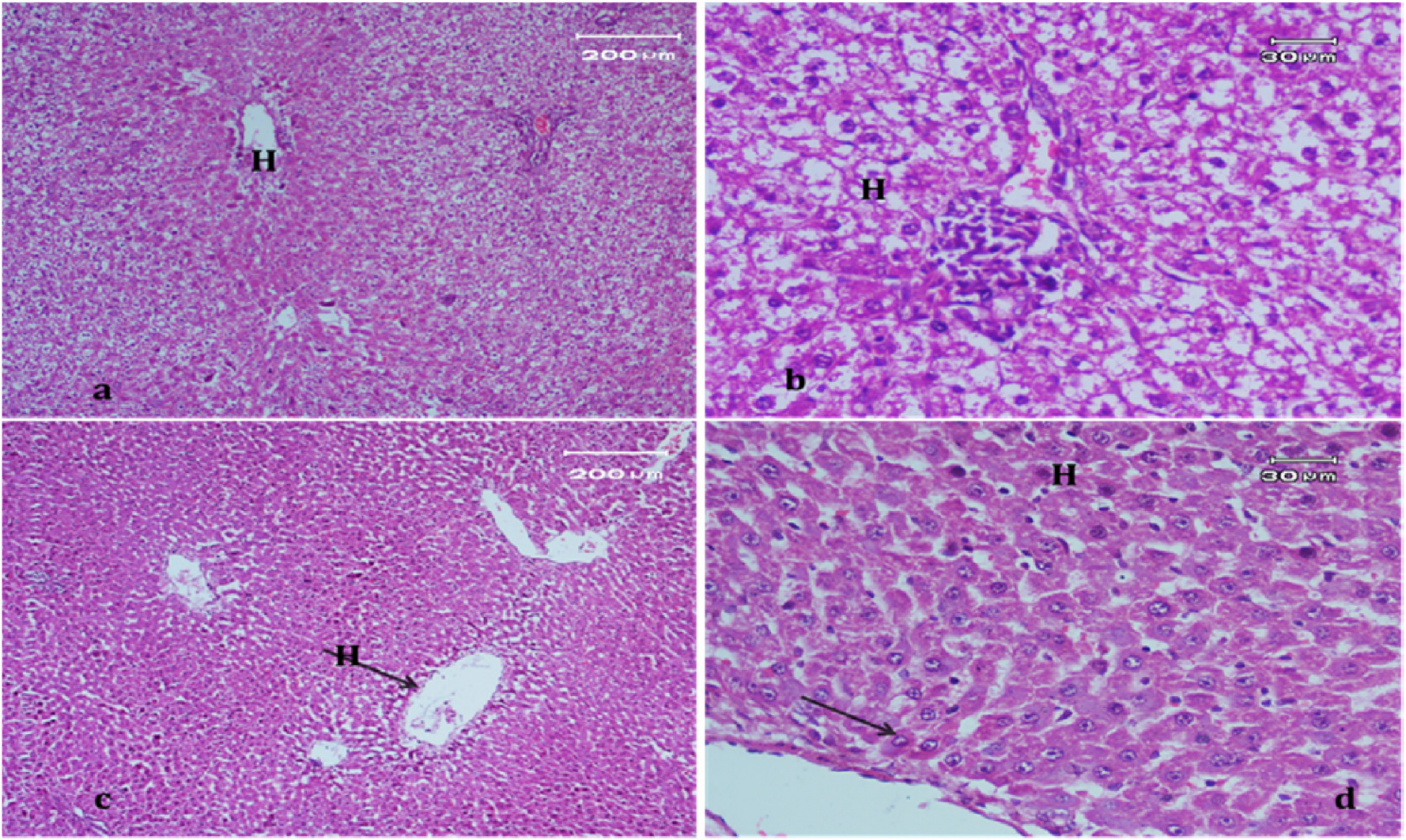
Light micrograph of a rat liver tissue treated with Low dose of butanol citrus extract (a,b) showing marked improvement in hepatocellular architecture and high dose (c&d) (H&E. 100 × 400).

**Figure 7:**
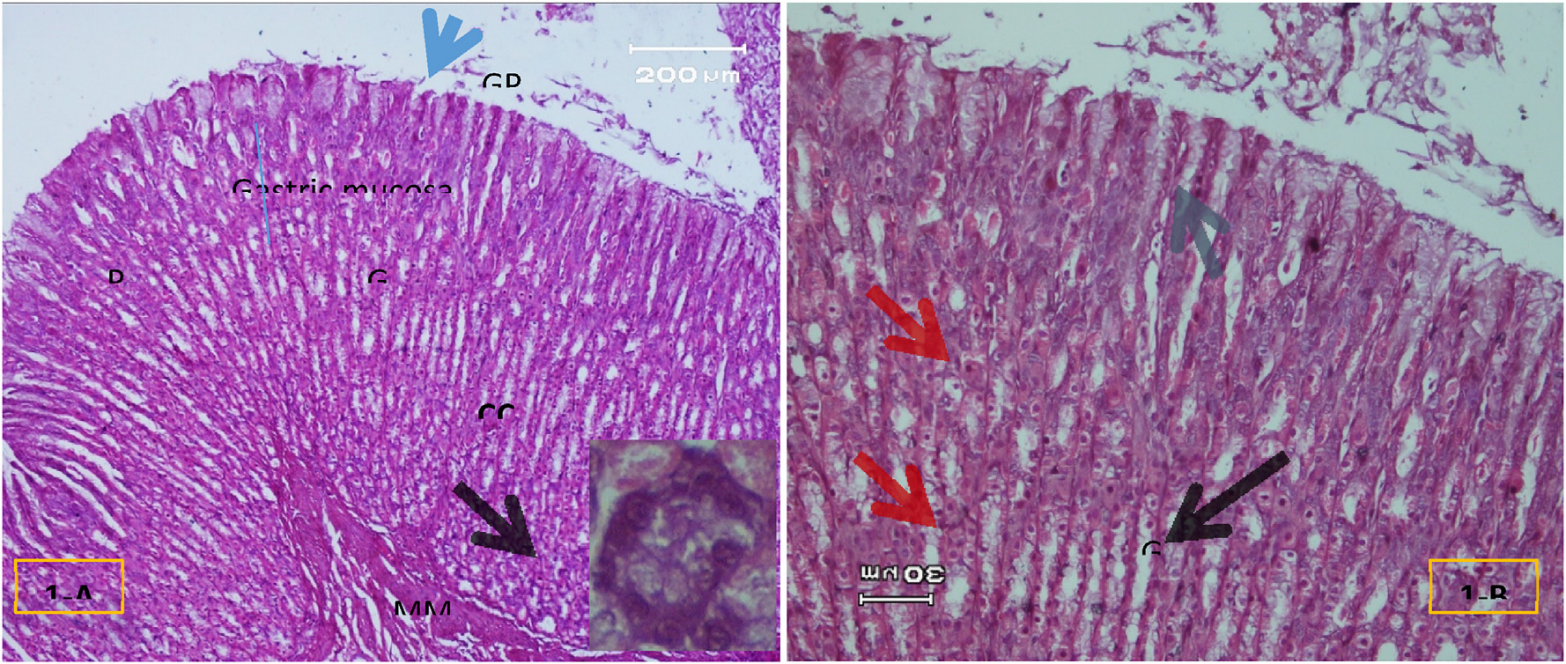
Negative control group (H&E. X 100,200,400).

**Figure 8:**
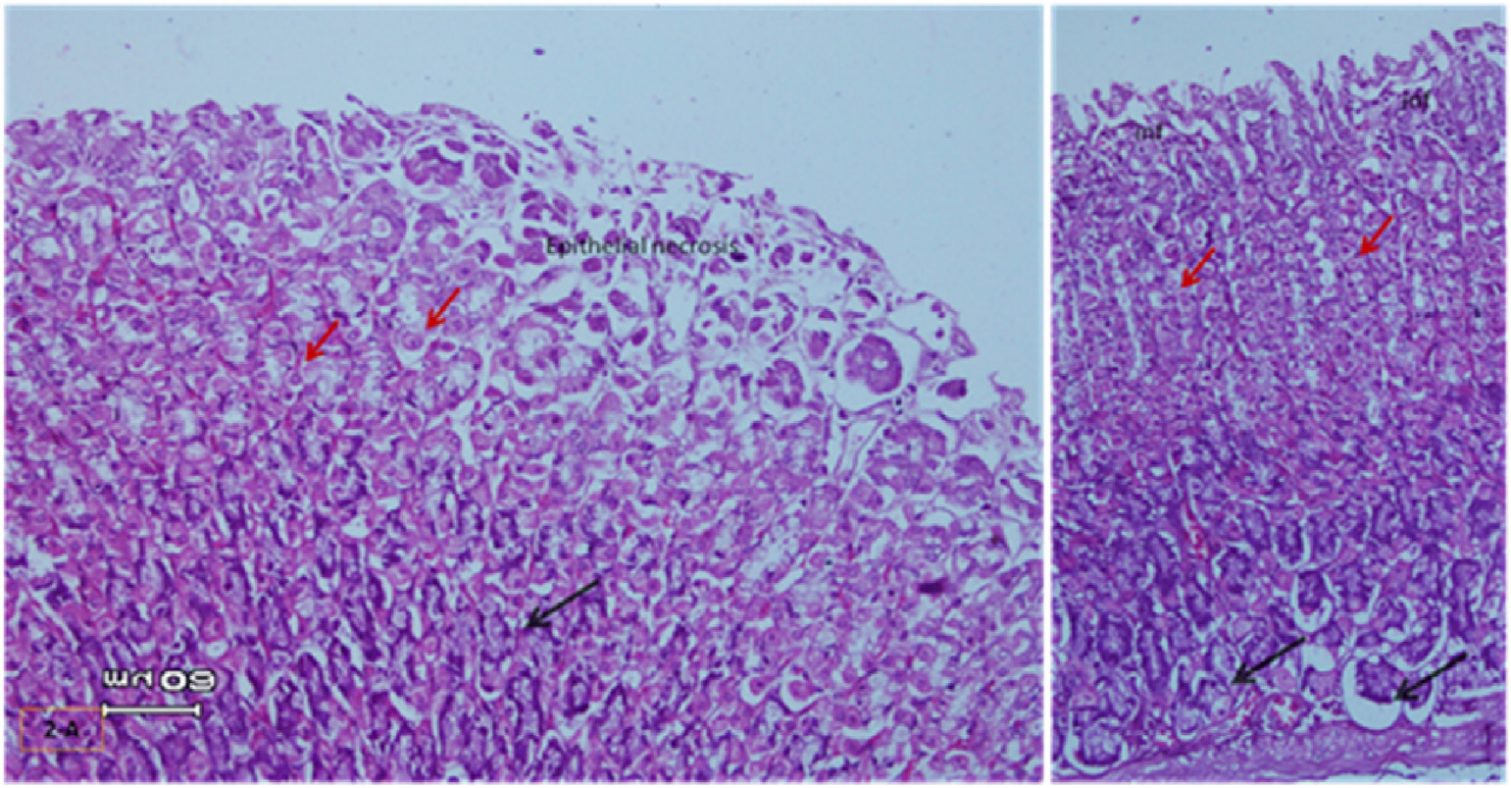
Positive control (H&E. X 100,200).

**Figure 9:**
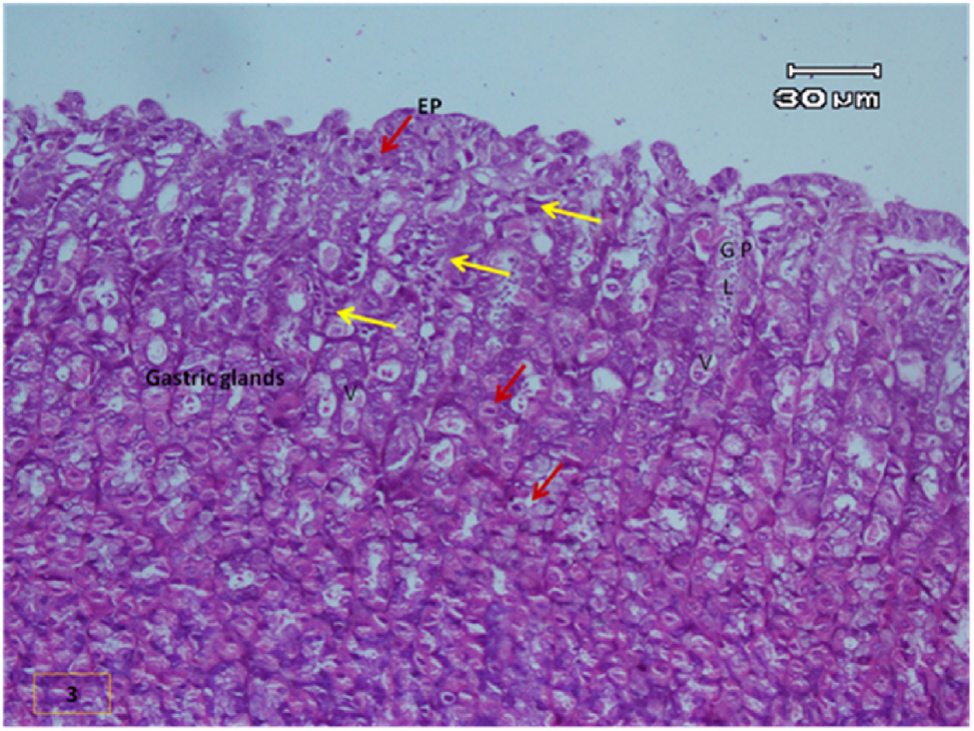
Stomach of reference group treated with ranitidine (H&E.X200).

The light micrograph of a rat liver tissue in the treated groups revealed the following findings. The reference group (Figure 3) treated with silymarin (25 mg/kg) before the administration of paracetamol showed focal necrotic cells around congested blood vessels. Rats treated with low dose of aqueous citrus extract (Figure 4a and b) exhibited severe vacuolar degeneration around the central vein, congested and fibrosed portal (arrow head) with inflammatory cells (arrow), and hypertrophy of the bile duct. However, rats treated with high dose (Figure 4c and d) showed marked improvement in hepatic histological features with vesiculated nuclei and mild dilatation in blood sinusoids. The light micrograph of a rat liver tissue treated with low dose of hesperidin (Figure 5a and b) revealed massive alteration in the liver parenchyma and necrosis randomly distributed throughout the parenchyma, massive vacuolar degeneration (V), and periportal inflammation (inf). However, rats treated with high dose of hesperidin (Figure 5c and d) exhibited marked improvement in hepatic histological features, with vesiculated nuclei and prominent dilatation in blood sinusoids and focal nuclear pyknosis in the middle of the lobule (red arrow). The light micrograph of a rat liver tissue treated with low dose of butanol citrus extract (Figure 6a and b) revealed marked improvement in the hepatocellular architecture, and for high-dose treatment (Figure 6c and d), the light micrograph revealed focal vacuolar degeneration around the central vein (arrow). Photomicrographs of the stomach of negative control rats (Figure 7) revealed normal histological structure of gastric mucosa consisting of surface epithelium, gastric pits, gastric glands, lamina propria, and muscularis mucosa (MM). The lamina propria was occupied with simple branched tubular adjacent glands (GG), which were lined by mucous neck cells (blue arrow), parietal cells (red arrow), and peptic cells (CC) in set. Photomicrographs of the stomach of positive control rats treated with 1 ml of 70%e thanol (Figure 8) revealed extensive gastric mucosal necrosis, ulceration, and excess cellular debris with inflammatory cell infiltration (inf). The submucosal secreting cells showed vascular degeneration with pyknotic nuclei (red arrow). Some chief cells were damaged or shrunken with congestion in the lamina propria. In contrast, the photomicrographs of the stomach of the reference group animals treated with ranitidine (Figure 9) revealed mild atrophy and interruption in the superficial epithelial cells of mucosa with pyknotic nuclei. Foci of inflammatory cells (yellow arrows) and leukocytes (L).

**Figure 10:**
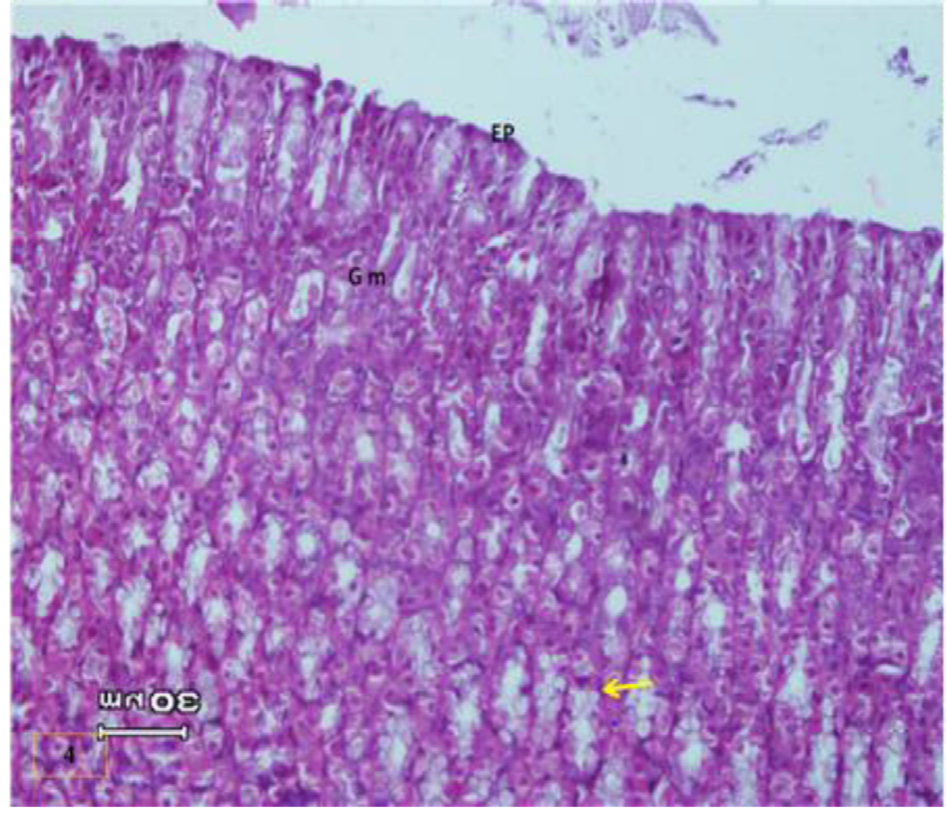
Stomachs of rats treated with low and high doses of aqueous extracts of citrus respectively (H&E. X 200).

**Figure 11:**
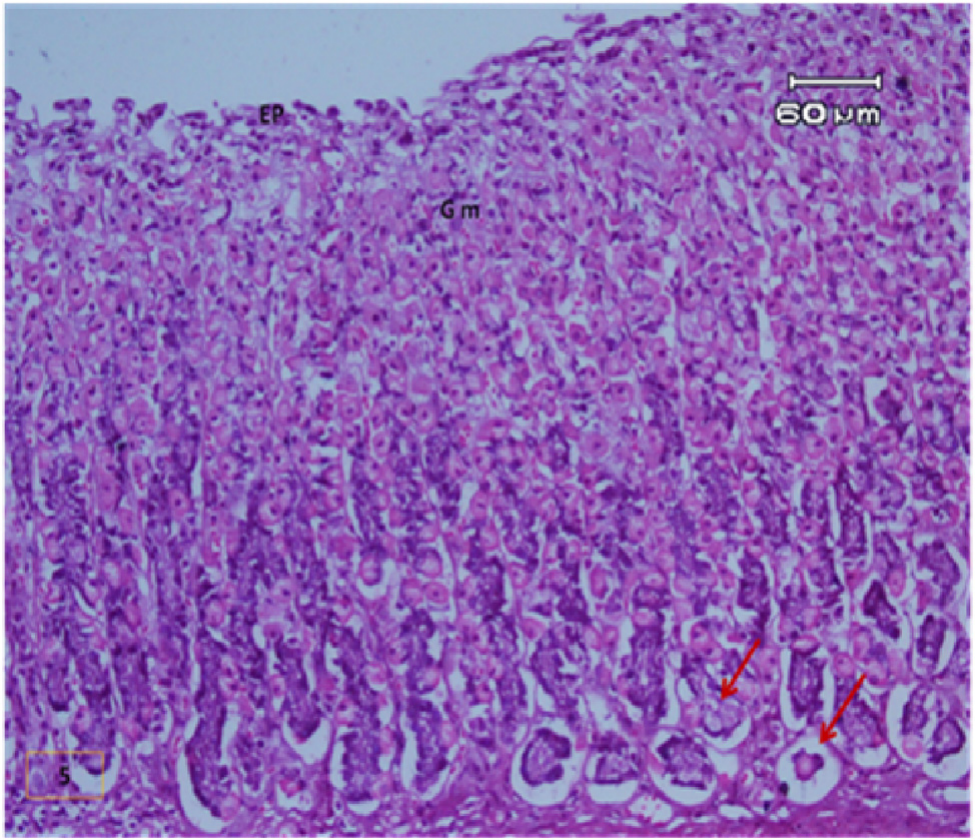
Stomachs of rats treated with low and high doses of aqueous extracts of citrus respectively (H&E. X 200).

**Figure 12:**
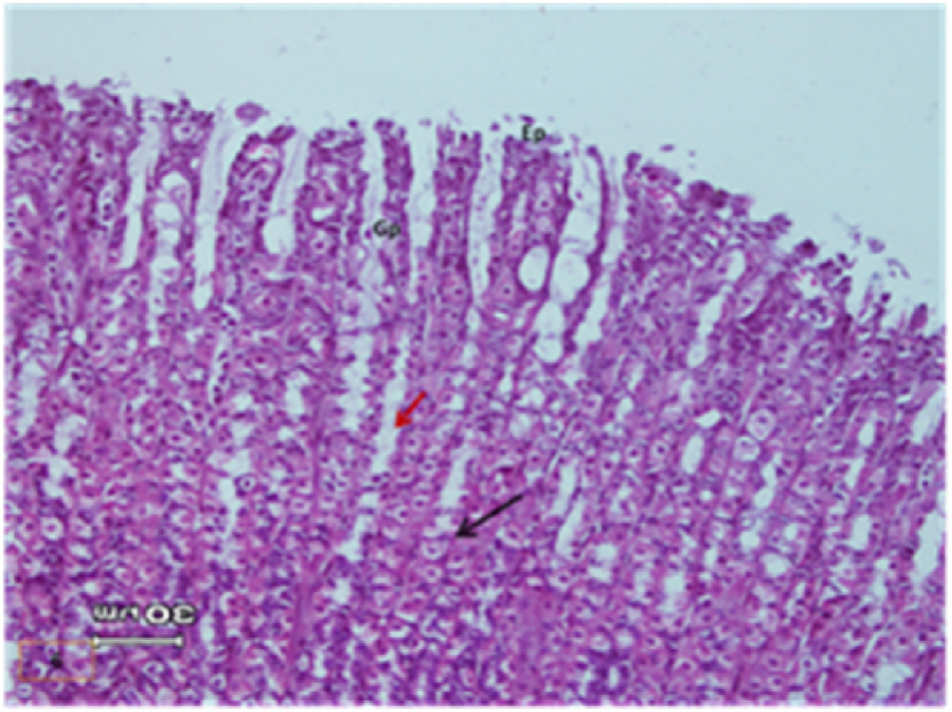
Stomachs of rats treated with low & high doses of butanol citrus extract (H&E. X 200).

**Figure 13:**
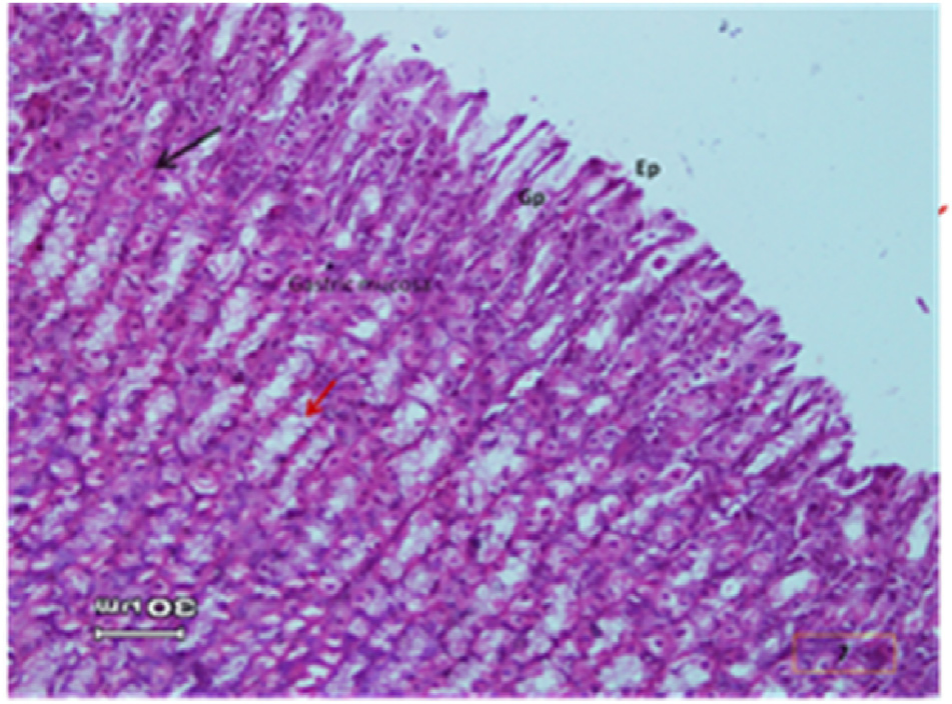
Stomachs of rats treated with low & high doses of butanol citrus extract (H&E. X 200).

**Figure 14:**
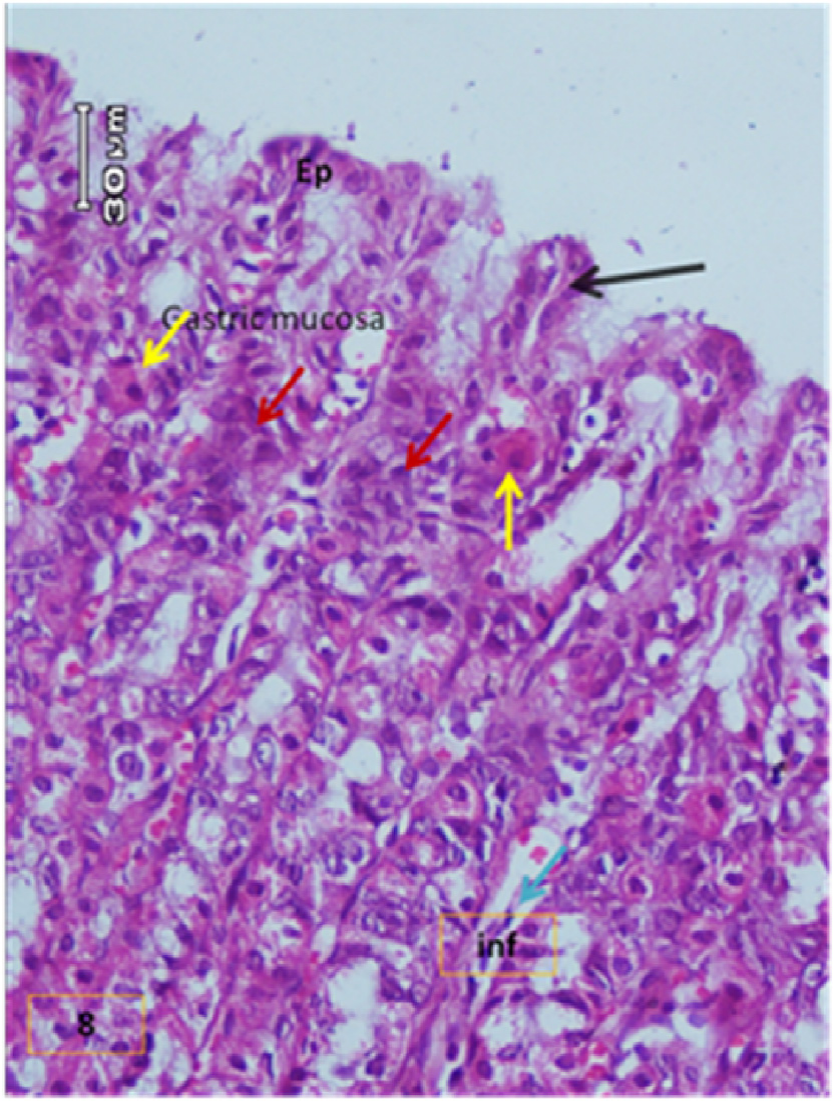
Stomachs from rats treated with low and high doses of hesperidin (H&E. X 400).

**Figure 15:**
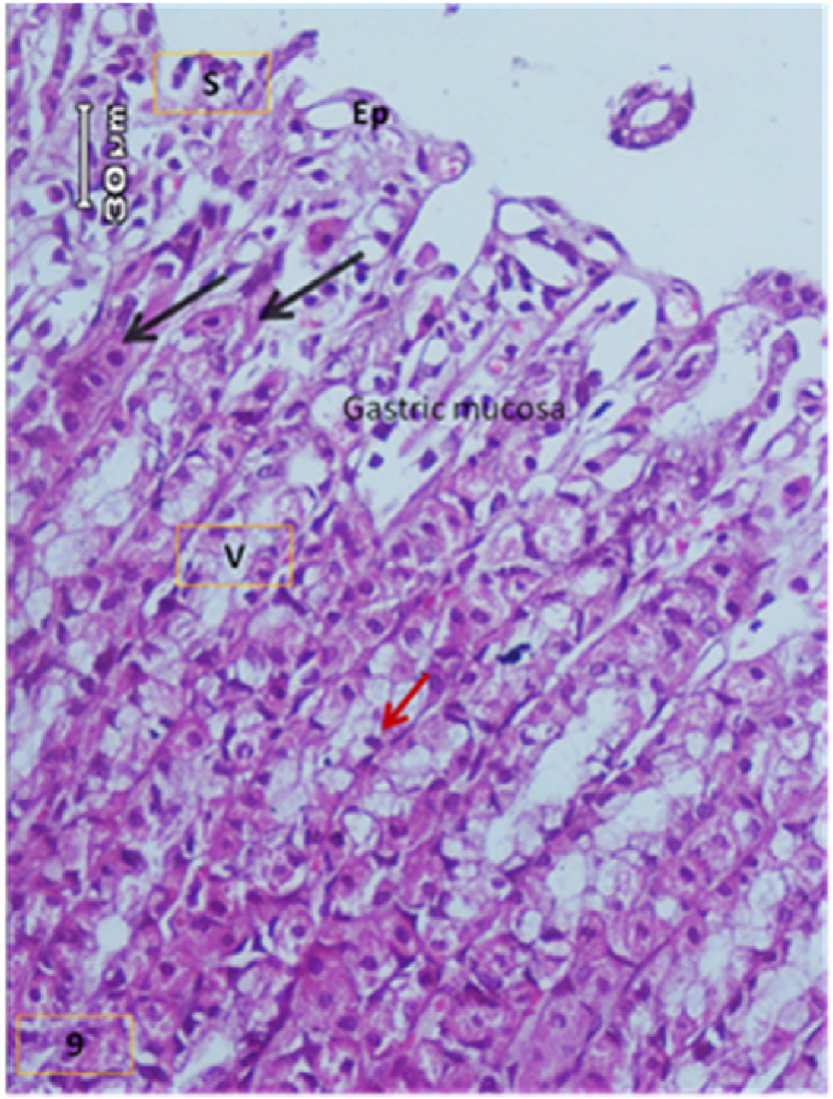
Stomachs from rats treated with low and high doses of hesperidin (H&E. X 400).

The gastric glands were lined with epithelium exhibiting cellular crowding, swelling, and loss of cell boundaries with nuclear pleomorphism. Some parietal cells exhibited vacuolation of cytoplasm (V), and other cells exhibited pyknotic nuclei (red arrows). Figure 10 shows the photomicrograph of the stomach of rats treated with low dose of citrus aqueous extract, which revealed preservation of mucosal architecture (crypts and gastric glands), intact gastric pits, and almost normal secreting glands with normal gastric mucosal cells (yellow arrow). Figure 11 shows the photomicrograph of the stomach of rats treated with high dose of citrus aqueous extract, which revealed distortion of mucosal epithelial cell lining with inflammatory cell infiltration. Some parietal cells were swollen and more accumulated at the base of glands. Necrotic chief cells with vacuolated cytoplasm and pyknotic nuclei were also observed (red arrow). In the butanol citrus extract treatment, the photomicrograph of the stomach of rats treated with low dose of the extract revealed wide areas of epithelial ulceration, superficial necrosis with inflammatory cell infiltration, induced dilation, irregular gastric pits, desquamation of mucosal cells (red arrow), and complete vacuolar cytoplasm of parietal cells (Figure 12). The photomicrograph of the stomach of rats treated with high dose of the extract revealed regular arrangement and intact superficial epithelial mucosa and gastric pits. Most secreting cells were almost normal with regular gastric glands with dilated lumina (red arrow, Figure 13). In the hesperidin treatment, the photomicrograph of the stomach of rats treated with low dose revealed preservation of mucosal structure (crypts and gastric glands) and foci of inflammatory cells (collected fusiform cells). Most secreting cells of gastric glands were damaged with pyknotic nuclei and apoptosis (red and yellow arrows, Figure 14). The photomicrograph of the stomach of rats treated with high dose of hesperid in showed partial loss of surface epithelium with sloughed areas (S) and widespread atrophy in mucosal epithelial cells and gastric pits (Figure 15). The dilated gastric glands were lined with necrotic and vacuolated cells with pyknotic nuclei (red arrow) that encroached on the lumen of gastric glands.

**1. Histopathology results**

The light micrograph of a negative control rat liver tissue (Figure 1) revealed normal liver hepatocytes and cords radiating from the central vein with blood sinusoids in between (BS) with rounded vesicular nuclei and granular cytoplasm (black arrow). The light micrograph of a positive control rat liver tissue treated with paracetamol (640 mg/kg) only (Figure 2) revealed periportal necrosis, fibrosis, inflammatory cell infiltration, bands of connective tissues (inf), and hypertrophy of portal triad and bile duct (red arrow). The light micrograph of a rat liver tissue from the reference group (Figure 3) treated with silymarin (25 mg/kg) before paracetamol administration revealed focal necrotic cells around congested blood vessels. The light micrograph of a rat liver tissue with low-dose citrus aqueous extract treatment (Figure 4a and b) revealed severe vacuolar degeneration around the central vein and congested and fibrosed portal (arrow head) with inflammatory cells (arrow) and hypertrophied bile duct. Rats treated with high-dose citrus aqueous extract (Figure 4c and d) showed marked improvement in hepatic histological features with vesiculated nuclei and mild dilatation in blood sinusoids. The light micrograph of a rat liver tissue with low-dose hesperidin treatment (Figure 5 a and b) revealed massive alteration in the liver parenchyma and necrosis randomly distributed throughout the parenchyma, massive vacuolar degeneration (V), and periportal inflammation (inf). However, rats treated with high-dose hesperidin (Figure 5c and d) exhibited marked improvement in hepatic histological features, with vesiculated nuclei and prominent dilatation in blood sinusoids focal nuclear pyknosis in the middle of the lobule (red arrow). The light micrograph of a rat liver tissue under low-dose butanol citrus extract treatment (Figure 6a and b) revealed marked improvement in the hepatocellular architecture, and that of rat liver tissue under high-dose treatment (Figure 6c and d) revealed focal vacuolar degeneration around the central vein (arrow).

**2. In vivo antioxidant activity of citrus aqueous and butanol extracts and hesperidin (125 and 250 mg/kg) in rats with experimentally induced hepatotoxicity using paracetamol (640 mg/kg)**

Results are expressed in Table 4.

**3. In vivo antioxidant activity of citrus aqueous and butanol extracts and hesperidin (125 and 250 mg/kg) in rats with experimentally induced gastric ulcers using ethanol (70%, 1 ml/rat). Ranitidine (50 mg/kg) was used as a reference drug. Results are expressed in**Table 5.

##### 3.2.2.6 Anti-inflammatory activity and antiulcerogenic effect of the two citrus extracts and hesperidin

**1. Macroscopic examination of gastric mucosa**

**Table 5:** Antioxidant activity of the two citrus extracts and hesperidin in rat models of gastric ulceration.

Group	Parameter		
	No (μmol/g Tissue)	CAT (μ/g Tissue)	MDA (nmol/g tissue)
Negative Control	10 ± 0.4	3.4 ± 0.11	60.5 ± 1.9
Positive Control group Ethanol 70% (1ml/rat)	46.8 ± 3.92^@^	0.95 ± 0.02^@^	173.7 ± 2.42^@^
Reference group Ranitidine (50 mg/kg)	15.8 ± 0.63∗	2.8 ± 0.05∗	67.1 ± 0.46∗
Aqueous citrus extract (125 mg/kg)	16.7 ± 0.34∗	2.35 ± 0.14^@^∗	92.55 ± 1.93^@^∗^$^
Aqueous citrus extract (250 mg/kg)	14.9 ± 0.63∗	2.85 ± 0.86^@^∗	78.25 ± 0.49^@^∗^$&^
Butanol citrus extract (125 mg/kg)	15 ± 1.09∗	2.5 ± 0.05^@^∗^!#^	81.6 ± 1.38^@^∗^$&#^
Butanol citrus extract (250 mg/kg)	16 ± 1.32∗	3.2 ± 0.05∗^&#£^	59.68 ± 1.37∗^&ˆ!£^
Hesperidin (125 mg/kg)	15.85 ± 0.72∗	3.6 ± 0.17∗^$&ˆ^	76.65 ± 1.35^@^∗^$&^
Hesperidin (250 mg/kg)	11.3 ± 0.23∗	4.55 ± 0.14^@^∗^$&ˆ^	67.75 ± 0.77^@^∗^$&ˆ!^

Results are expressed as mean of levels + S.E of NO, CAT and MDA in stomach homogenates of rats treated with aqueous citrus extract, hesperidin, and butanol citrus extract (125 and 250 mg/kg) and ranitidine (50 mg/kg) for two successive weeks followed by induction of gastric ulcers by using ethanol 70% 1 ml/rat; n = 8; Data were analysed using one way analysis of variance (ANOVA) followed by Tukey Kramer multiple comparisons test; Significant at P < 0.05@ Significant different from negative control; ∗Significant difference from positive control group; $Significant difference from Ranitidine group; &Significant difference from the aqueous citrus extract (125 mg/kg) group; ˆSignificant difference from the aqueous citrus extract (250 mg/kg) group; !Significant difference from the hesperidin (125 mg/kg) group; #Significant difference from the hesperidin (250 mg/kg) group; £Significant difference from the butanol citrus extract (125 mg/kg) group.
**2. Biochemical results of anti-inflammatory activity in the gastroprotective**

**Table 6:** The effects of the two citrus extracts and hesperidin on gastric ulcer No. and severity.

Group	Parameter	
	Number of Ulcer	Severity of Ulcers
Negative Control	–	–
Positive control group Ethanol 70%	4 ± 0.4^@^	3.5 ± 0.45^@^
Reference group Ranitidine (50 mg/kg)	1.16 ± 0.08^@^∗	0.66 ± 0.08∗
Aqueous citrus extract (125 mg/kg)	1 ± 0.03∗	1 ± 0.05∗
Aqueous citrus extract (250 mg/kg)	2 ± 0.2^@^∗	1 ± 0.14∗
Hesperidin (125 mg/kg)	1.33 ± 0.09^@^∗	1.33 ± 0.16^@^∗
Hesperidin (250 mg/kg)	2 ± 0.28^@^∗	2.66 ± 0.33^@$&ˆ!^
Butanol citrus extract (125 mg/kg)	0.33 ± 0.04 ∗^ˆ#^	0.33 ± 0.08∗^#^
Butanol citrus extract (250 mg/kg)	0.66 ± 0.08∗^ˆ#^	0.33 ± 0.08∗^#^

Results are expressed as mean of levels + S.E of number and severity of ulcers of stomachs of rats treated with hesperidin, aqueous and butanol citrus extracts (125 and 250 mg/kg) and ranitidine (50 mg/kg) for two successive weeks followed by induction of gastric lesions by using ethanol 70% (1 ml); n = 8; Data were analysed using one way analysis of variance (ANOVA) followed by Tukey Kramer multiple comparisons test; Significant at P < 0.05.

@ Significant difference from negative control; ∗Significant difference from positive control group; $ Significant difference from ranitidine group; &Significant difference from the aqueous citrus extract (125 mg/kg) group; ˆSignificant difference from the aqueous citrus extract (250 mg/kg) group; ! Significant difference from hesperidin (125 mg/kg) group; # Significant difference from hesperidin (250 mg/kg) group.
**3. Histopathological results on the effect of citrus aqueous and butanol extracts and hesperidin on gastric mucosa**

**Table 7:** Effects of the two citrus extracts and hesperidin on inflammatory markers.

Groups	Parameters	
	CRP (ng/ml)	Ilβ6 (pg/ml)
Negative Control	1.36 ± 0.08	45.86 ± 2.49
Positive control group Ethanol 70% (1ml/rat)	3.45 ± 0.3^@^	103.8 ± 3.27^@^
Reference group Ranitidine (50 mg/kg)	2.2 ± 0.02^@^∗	69.67 2.09^@^∗^$^
Aqueous citrus extract (125 mg/kg)	2.12 ± 0.13^@^∗	69.67 ± 2.09^@^∗^$^
Aqueous citrus extract (250 mg/kg)	1.9 ± 0.17∗	52.6 ± 1.55∗^&^
Hesperidin (125 mg/kg)	2.39 ± 0.06 ^@^∗	52.03 ± 1.7∗^&^
Hesperidin (250 mg/kg)	1.83 ± 0.15∗	41.67 ± 2.56∗^&^
Butanol citrus extract (125 mg/kg)	2.42 ± 0.09 ^@^∗	60.33 ± 3.12^@^∗^$#^
Butanol citrus extract (250 mg/kg)	1.95 ± 0.1∗	49.88 ± 3.66∗^&^

Results are expressed as mean of levels of CRP and IL 136 + S.E in serum of rats treated with aqueous citrus extract, hesperidin, and butanol citrus extract (125 and 250 mg/kg) and ranitidine (50 mg/kg) for two successive weeks followed by induction of gastric lesions by using ethanol 70% (1 m1); n = 8; Data were analysed using one way analysis of variance (ANOVA) followed by Tukey Kramer multiple comparisons test; Significant at P < 0.05 @ Significant different from negative control; ∗Significant difference from positive control group; group; $ Significant difference from ranitidine group; &Significant difference from the aqueous citrus extract(125 mg/kg) group; ˆSignificant difference from the aqueous citrus extract (250 mg/kg) group; ! Significant difference from the hesperidin (125 mg/kg) group; # Significant difference from the hesperidin (250 mg/kg) group.

The photomicrographs of the stomach of negative control rats (Figure 7) revealed normal histological structure of gastric mucosa consisting of surface epithelium, gastric pits, gastric glands, lamina propria, and MM. The lamina propria was occupied with simple branched tubular adjacent glands (GG), which were lined by mucous neck cells (blue arrow), parietal cells (red arrow), and peptic cells (CC) in set. The photomicrographs of positive control rats treated with 1 ml of 70% ethanol (Figure 8) revealed extensive gastric mucosal necrosis, ulceration, and excess cellular debris with inflammatory cell infiltration (inf). The submucosal secreting cells exhibited vascular degeneration with pyknotic nuclei (red arrow). Some chief cells were damaged or shrunken with congestion in the lamina propria. In contrast, the photomicrographs of the stomach of the reference group animals treated with ranitidine (Figure 9) showed mild atrophy and interruption in the superficial epithelial cells of mucosa with pyknotic nuclei. Foci of inflammatory cells (yellow arrows) and leukocytes (L).

The gastric glands were lined with epithelium exhibiting cellular crowding, swelling, and loss of cell boundaries with nuclear pleomorphism. Some parietal cells exhibited vacuolation of cytoplasm (V), whereas other cells exhibited pyknotic nuclei (red arrows). In Figure 10 the photomicrograph of the stomach of rats treated with low dose of aqueous extract of citrus shows preservation of mucosal architecture (crypts and gastric glands), intact gastric pits, the secreting glands are nearly normal gastric mucosal cells (yellow arrow). While in Figure 11; photomicrograph of the stomach of rats treated with high dose of the aqueous extract of citrus shows distortion of mucosal epithelial cell lining which infiltrated by inflammatory cells, Some parietal cells are swollen and more accumulated at the base of the glands. Necrotic chief cells with vacuolated cytoplasm and pyknotic nuclei are also seen (red arrow). Regarding the butanol citrus extract; the photomicrograph of the stomach of a rat treated with low dose of the extract shows wide areas of epithelial ulceration superficial necrosis infiltrated with inflammatory cells induced dilating and irregular gastric pits and desquamation of mucosal cells (red arrow) complete vacuolar cytoplasm of parietal cells (Figure 12), while a photomicrograph of the stomach of a rat treated with high dose of the extract shows regular arrangement and intact superficial epithelial mucosa and gastric pits. Most of secreting cells are nearly normal. regular gastric glands with dilated lumina (red arrow, Figure 13). As for the hesperidin; photomicrograph of the stomach from rat treated with low of the extract shows preservation of mucosal structure (crypts and gastric glands), foci of inflammatory cells (collected fusiform cells), Most of gastric glands secreting cells are damaged with pyknotic nuclei cells and apoptosis (red & yellow arrows, Figure 14), while in Figure 15; the photomicrograph of the stomach from rat treated with high dose of the extract shows partial loss of surface epithelium sloughed areas (S) and wide spread of atrophy in mucosal epithelial cells and gastric pits. The dilated gastric glands are lining with necrotic and vacuolated cells with pyknotic nuclei (red arrow) and encroached on the lumen of the gastric glands.

## 4 Discussion

This study was conducted to investigate the potential efficacy of butanol and aqueous citrus extracts and hesperidin to be used as promising anti-inflammatory, antioxidant, hepatoprotective, and gastroprotective natural supplements. The doses used in this study were 125 and 250 mg/kg. Their effects on the digestive system were examined using animal models of hepatotoxicity associated with oxidative stress induced using paracetamol (640 mg/kg) and compared with the effect of silymarin (25 mg/kg), which was used as a standard drug used for alleviating the signs and symptoms of hepatic insult in patients. In addition, their protective effects on the gastric mucosa against gastritis and ulceration associated with their antioxidant activity were investigated using animal models of gastric ulcer induced using 70% ethanol (1 ml/rat) and compared with the effect of ranitidine (50 mg/kg), which was used as a standard drug against gastritis.

The biochemical and histopathological results concerning the hepatoprotective effects of hesperidin and aqueous and butanol citrus extracts were concomitant with each other. Paracetamol (640 mg/kg) induced hepatotoxicity manifested by increased levels of hepatic enzymes; it also exerted an oxidative effect as evidenced by increased MDA and NO levels and decreased CAT levels. Moreover, light microscopic examination revealed abnormal hepatic architecture. In contrast, silymarin, which was used as a reference drug, and the three tested agents exhibited reduction of hepatic enzyme levels and antioxidant activity, which was manifested by reduced MDA and NO levels and increased CAT levels and improved the hepatic architecture, as observed in the histopathological examination.

Regarding the gastroprotective effects, we found that all the experimental results concerning the evaluation of antioxidant, anti-inflammatory, and subsequently gastroprotective activities, which were confirmed by macroscopic and microscopic examinations of the gastric mucosa dissected from all the tested animal groups, were concomitant with each other.

The damaging effect of ethanol in our study is due to its ability to cause oxidative stress and high inflammatory activity, evidenced by increased MDA, NO, CRP, and IL-β6 levels and decreased CAT levels. Ibrahim et al. (2016a, 2016b, 2016c) reported that ethanol decreases bicarbonate secretion and gastric wall mucus and increases capillary permeability, subsequently causing edema followed by gastric wall ulceration.

In contrast, the reference antiulcerogenic drug ranitidine, besides being an H_2_ receptor antagonist that decreases acid production, exerted significant antioxidant and anti-inflammatory effect, as manifested by reduction of MDA, NO, CRP, and IL-β6 levels and increased CAT levels. A similar finding was also reported by Ahmadi et al. (2011) who confirmed that ranitidine exhibited antioxidant and antinociceptive activities.

The three tested agents in this study exerted significant antioxidant, anti-inflammatory, and digestive system protective effects against malicious insult by chemicals, such as the drug-induced hepatotoxicity by paracetamol or gastritis and subsequently ulceration induced by ethanol.

These protective effects of hesperidin and aqueous and butanol citrus extracts may be attributed to their phytochemical nature as they are rich in phenolic acids and flavonoids, which influenced their mechanisms of action and enhanced their effects through antioxidant and anti-inflammatory processes.

The major bioactive phenols are phenolic acids, flavonoids, stilbenes, lignans, and tannins. Phenols exert antioxidant activity through hydrogen donation of the phenolic hydroxyl group (Blomhoff 2010).

HPLC analysis revealed the presence of phenolic acids and flavonoids in our study, such as hesperidin, apigenin, glucosides, and quercetin.

The HPLC analysis revealed the presence of hesperidin in the aqueous and butanol citrus extracts at concentrations of 1526.7 and 2086.7 μg/g, respectively.

This HPLC finding explains the hepatoprotective, gastroprotective, antioxidant, and systemic anti-inflammatory effects of both aqueous and butanol citrus extracts, in addition to the isolated hesperidin compound used in this study. These protective effects due to the antioxidant capacity of hesperidin have been previously explained by Parhiz et al. (2015), who reported that the radical scavenging mechanism of action of the phenolic compound hesperidin is through the ERK/Nrf 2 signaling pathway. Moreover, Asjad et al. (2013) showed that the antioxidant capacity of hesperidin extracted from *C. sinensis* peels was 36%. In addition to its antioxidant activity, Roohbakhsh et al. (2015) reported that hesperidin demonstrated anti-inflammatory activities in their study.

Aboul Naser et al. (2020) also attributed the protective effects of hesperidin to its phytochemical nature as flavanone and being a phenolic compound, which strengthen our conclusion that the hepatoprotective and gastroprotective effects in our study are due to the presence of hesperidin in all the tested agents and its action as an antioxidant and anti-inflammatory substance. Ahmad et al. (2012) mentioned that hesperidin (3, 5, 7-trihydroxy flavanone-7-rhamnoglucoside) exerted potent antihepatotoxic effects against paracetamol-induced toxicity, and Pari et al. (2015) reported that it exhibited high antioxidant activity and free radical scavenging effect against oxidative stress. Moreover, Sam-Long et al. (2008) demonstrated good intracellular free radical scavenging activity associated with the ability to inactivate reactive metabolites and reactive oxygen species at their production site of hesperidin its hydrophilic glycosidic component that maintains its presence in the cellular cytoplasm for long periods of time, so as what Leelavinothan et al. (2015), have mentioned, it could protect the endogenous antioxidant enzymes. Hence, the hepatoprotective effects of both hesperidin and aqueous or butanol citrus extracts can be attributed to their strong antioxidant capacity that aided in preventing the peroxidation of polyunsaturated lipids found in the plasma membrane, endoplasmic reticulum, and mitochondria, helping in the maintenance of their integrity and reducing the levels of hepatic enzymes.

In addition to the presence of hesperidin in the aqueous and butanol extracts in our study, we detected other compounds in considerable amounts such as quercetin, apigenin 7-glucoside, chlorogenic acid, syringic acid, para coumaric acid, and rutin in the butanol extract and apigenin 7-glucoside, chrysin, p-coumaric acid, *p*-hydroxybenzoic acid, and gallic acid in the aqueous extract, which may explain the high protective ability against hepatotoxicity and gastric ulcer in our study. In particular, Alam et al. (2017) showed that rutin, quercetin, and gallic acid exerted a compacting effect on hepatitis B virus (HBV), describing that their hepatoprotective effects could be due to their antioxidant and anti-inflammatory activities, which were also obvious in our study results. Furthermore, they mentioned that those compounds had the ability to inhibit HBV gene expression and DNA replication and exerted anticancer effects. In addition, Sowmya et al. (2016) mentioned that quercetin, which is a natural flavonol present in several plants, is used to treat hypercholesterolemia, cardiovascular diseases, peptic ulceration, and inflammation. Moreover, they stated that rutin, which is an intermediate glycoside between the flavonol quercetin and the disaccharide rutinose and is a phenolic compound, possesses anti-inflammatory activity. Furthermore, they mentioned about the antioxidant properties of gallic acid, which could explain the hepatoprotective and gastroprotective effects of both types of extracts in this study. In addition to these previous data, Tyśkiewicz et al. (2019) showed that the high ability to heal wounds and the antimicrobial effect of propolis and other natural products are due to the antioxidant effects of natural flavonoids, apigenin, and quercetin and kaempferol in small amounts, and this could also be a primary reason for their hepatoprotective and gastroprotective effects observed in our study.

We determined the gastroprotective effect associated with anti-inflammatory and antioxidant efficacy using animal models of gastric ulcer induced using 70% ethanol (1 ml/rat) and compared it with the effect of ranitidine (50 mg/kg), a standard drug used for the treatment of gastric ulcer in patients.

Our study demonstrated that all the three citrus agents exerted significant lowering effect on hepatic enzyme levels compared to that in the hepatotoxic group. High doses of all the tested agents exerted better effects than low doses; however, the effect was not dose-dependent. The best effects were exerted by hesperidin and butanol extract administered at high doses, as the AST levels were 31.93 and 28.75 U/L, respectively, and the ALT levels were 20.68 and 21.23 U/L, respectively. The results of biochemical parameters were consistent with those of the histopathological study. Regarding their antioxidant effects, the two extracts and hesperidin exerted significant antioxidant effects in the hepatotoxic model, which was evident by the significant reduction of NO and MDA levels and elevation of CAT levels compared to those in the hepatotoxic group wherein oxidative stress was induced using paracetamol (640 mg/kg). Moreover, high doses of all the tested agents exerted better effects than low doses, but this effect was not dose-dependent. The best effects were exerted by hesperidin and butanol extract administered at high doses, wherein the levels of NO were 10.88 and 9.97 μmol/g, respectively, and those of CAT were 5.2 and 3.47 μ/g, respectively. The best effects on MDA levels were obtained with aqueous and butanol extracts administered at high doses (65.3 and 59.68 nmol/g, respectively).

In the gastroprotective study, all the three agents resulted in a significant decrease in the number of gastric ulcers in the macroscopic examination of stomachs extracted from groups treated before the administration of 70% ethanol (l ml/rat) compared to those of the untreated group that received only 70% ethanol (1 ml/rat) without prior treatment. The effects of low doses were better than those of high doses. Their effects were better than those of ranitidine (50 mg/kg). The butanol extract at low dose exhibited the best result, as the number and severity of ulcers were 0.33 and 0.33, respectively, compared to those in the positive control (4 and 3.5) and those of ranitidine (1.16 and 0.66). The macroscopic examination results were consistent with those of the histopathological study. Regarding their antioxidant effects also, all the three agents exerted significant antioxidant effects in the gastric ulcer model, which were evident by the significant reduction of NO and MDA levels and elevation of CAT levels compared to those in the ulcer group wherein oxidative stress was induced using 70% ethanol (1 ml/rat). High doses of both aqueous extract and hesperidin exerted better effects than low doses on NO levels (14.9 and 11.3 μmol/g, respectively) and CAT levels (2.85 and 4.55 μ/g, respectively) compared with the positive control group (3.45) and ranitidine group (2.2) compared to positive control group 3.45 respectively and MDA levels (78.25 and 78.25 nmol/g) respectively, while the low dose of butanol extract had better effect on NO:16 μmol/g, but the effect of high dose was better on CAT:3.2 μ/g and MDA:64.6 nmo Ug.

The biochemical results concerning the anti-inflammatory effects of aqueous and butanol citrus extracts and hesperidin, low and high doses resulted insignificant anti-inflammatory activities, manifested by the reduction of CRP and IL-β6 levels in the serum of groups treated before the administration of 70% ethanol (1 ml/rat) compared with those of the untreated group that received only 70% ethanol (1 ml/rat) without prior treatment. The effect of high-dose hesperidin was the best, as the CRP level was 1.83 ng/ml compared with the positive control group (3.45 ng/ml) and ranitidine group (2.2 ng/ml), and the level of IL-β6 was 41.67 pg/ml compared with the positive control group (103.8 pg/ml) and ranitidine group (47.3 pg/ml).

Our results regarding the effect of butanol extract and hesperidin on inflammatory mediators were consistent with those reported by Motawi et al. (2012), Sabiu et al. (2014), Abd-Alla et al. (2016), Fahmy et al. (2019), and Aboul Naser et al. (2020). They stated that alcohol such as ethanol causes disturbances in gastric secretion that results in increased oxidative stress, lipid peroxidation, and dysfunction, leading to damage of cells.

## 5 Conclusion

Hesperidin exerted the best hepatoprotective, gastroprotective, antioxidant, and anti-inflammatory effects, followed by butanol extract and then aqueous extract in the present study. These protective effects of hesperidin are most probably because it is a phenolic compound and also due to the presence of phytochemicals such as flavanone.

This investigation, worldwide interest in natural products as preventive and therapeutic agent has given great appreciation of the rich culture heritage of traditional medicinal system. We used different wastes of citrus to add new source of these natural products isolated or recovered from wastes, such as seeds and peel of Navel orange and leaves originated from pruning process. This target makes use of these wastes in discovering new sources of therapeutic agents and get ride of the hazards induced by these wastes. HPLC analysis revealed the presence of phenolic acids and flavonoids, such as hesperidin, apigenin, glucosides, and quercetin.

## Declarations

### Author contribution statement

Doha H. Abou Baker; Bassant M.M. Ibrahim: Performed the experiments; Analyzed and interpreted the data; Contributed reagents, materials, analysis tools or data; Wrote the paper.

Yasmin Abdel-Latif: Analyzed and interpreted the data; Contributed reagents, materials, analysis tools or data; Wrote the paper.

Nabila S Hassan; Emad M. Hassan; Souad El Gengaihi: Conceived and designed the experiments; Wrote the paper.

### Funding statement

This study was supported by 10.13039/100007787National Research Centre [No. 11010318].

### Data availability statement

Data included in article/supp. material/referenced in article.

### Declaration of interest’s statement

The authors declare no conflict of interest.

### Additional information

No additional information is available for this paper.

## References

[bib1] Abd-Alla Howaida I. (2016). Phytochemical composition, protective and therapeutic effect on gastric ulcer and α-amylase inhibitory activity of Achillea biebersteinii Afan. Arch Pharm. Res..

[bib2] Abou Baker D.H., Rady Hanaa M. (2020). Bioassay-guided approach employed to isolate and identify anticancer compounds from *Physalis peruviana* calyces. Plant Arch..

[bib3] Abou Baker D.H., Al-Moghazy M., ElSayed A.A.A. (2020). The in vitro cytotoxicity, antioxidant and antibacterial potential of Satureja hortensis L. essential oil cultivated in Egypt. Bioorg. Chem..

[bib4] Abou Baker D.H., Ibrahim B.M., Hassan N.S., Yousuf A.F., El Gengaihi S. (2020). Exploiting *citrus aurantium* seeds and their secondary metabolites in the management of Alzheimer disease. Toxicol Rep.

[bib5] Aboul Naser Asmaa (2020). Management of Citrus sinensis peels for protection and treatment against gastric ulcer induced by ethanol in rats. Biomarkers.

[bib6] Ahmad S.T., Arjumand W., Nafees S., Seth A., Ali N., Rashid S., Sultana S. (2012 Jan 25). Hesperidin alleviates acetaminophen induced toxicity in Wistar rats by abrogation of oxidative stress, apoptosis and inflammation. Toxicol. Lett..

[bib7] Ahmadi A., Ebrahimzadeh M.A., Ahmad-Ashrafi S., Karami M., Mahdavi M.R., Saravi S.S. (2011). Hepatoprotective, antinociceptive and antioxidant activities of cimetidine, ranitidine and famotidine as histamine H2 receptor antagonists. Fund. Clin. Pharmacol..

[bib8] Alam P., Parvez M.K., Arbab A.H., Al-Dosari M.S. (2017 Jan 1). Quantitative analysis of rutin, quercetin, naringenin, and gallic acid by validated RP-and NP-HPTLC methods for quality control of anti-HBV active extract of Guiera senegalensis. Pharmaceut. Biol..

[bib58] Alkofahi A., Atta A.H. (1999). Pharmacological screening of the anti-ulcerogenic effects of some Jordanian medicinal plants in rats. J. Ethnopharmacol..

[bib9] Allam Sally Farouk (2018). How do mentha plants induce resistance against Tetranychus urticae (Acari: Tetranychidae) in organic farming?. J. Plant Protect. Res..

[bib10] Alvarez A. (1999). Gastric antisecretory and antiulcer activities of an ethanolic extract of Bidens pilosa L. var. radiata Schult. Bip. J. Ethnopharmacol..

[bib11] Asjad H.M., Akhtar M.S., Bashir S., Din B., Gulzar F., Khalid R., Asad M. (2013 Jan 13). Phenol, flavonoid contents and antioxidant activity of six common citrus plants in Pakistan. Journal of Pharmaceutical and Cosmetic Sciences.

[bib12] Abou Baker Doha (2020). Plants against Helicobacter pylori to combat resistance: an ethnopharmacological review. Biotechnol. Rep..

[bib13] Abou Baker Doha H. (2020). Achillea millefolium L. ethyl acetate fraction induces apoptosis and cell cycle arrest in human cervical cancer (HeLa) cells. Ann. Agric. Sci. (Cairo).

[bib14] Bancroft John D., Gamble Marilyn (2008). Theory and Practice of Histological Techniques.

[bib15] Bhandari Uma (2003). Antihepatotoxic activity of ginger ethanol extract in rats. Pharmaceut. Biol..

[bib16] Blomhoff R. (2010).

[bib17] Clementi Giuseppe (1998). Effects of centrally or peripherally injected adrenomedullin on reserpine-induced gastric lesions. Eur. J. Pharmacol..

[bib18] Drury R.A.B., Wallington E.A. (1980).

[bib19] Edwards-Jones Valerie (2004). The effect of essential oils on methicillin-resistant Staphylococcus aureus using a dressing model. Burns.

[bib20] El-gengaihi Souad E. (2020). Chemical, biological, and molecular studies on different citrus species wastes. Plant Arch.

[bib21] El-gengaihi S.E., Hamed M.A., Aboubaker D.H., Mossa A.T. (2016). Flavonoids from sugar beet leaves as hepatoprotective agent. Int. J. Pharm. Pharmaceut. Sci..

[bib22] El-gengaihi Souad (2016). Hepatoprotective efficacy of Cichorium intybus L. extract against carbon tetrachloride-induced liver damage in rats. J. Diet. Suppl..

[bib56] Fellers P.J. (1985).

[bib25] Hussein J.I., El-Matty D., El-Khayat Z.A., Abdel-Latif Y.A. (2012). Brain neurotransmitters in diabetic rats treated with CO enzyme Q10. Int. J. Pharm. Pharmaceut. Sci..

[bib26] Hwang E.S., Do Thi N. (2014). Effects of extraction and processing methods on antioxidant compound contents and radical scavenging activities of laver (Porphyra tenera). Prevent. Nutr. Food Sci..

[bib28] Ibrahim B.M., Yassin N.A., Hetta M.H., Ta K.F., Mohammed W.I., Hassan M.E. (2016). Phytochemical and pharmacological studies on newly-suggested herbal formulations for potential protection against inflammatory conditions. Int. J. Pharmacogn. Phytochem. Res..

[bib29] Ibrahim Bassant MM. (2016). Study of the protective effects of flaxseed oil on ethanol induced gastric mucosal lesions in non ovariectomized and ovariectomized rats. Int. J. Pharmacol..

[bib30] Ibrahim Eman A., Abou Baker Doha H., El-Baz Farouk K. (2016). Anti-inflammatory and antioxidant activities of rhubarb roots extract. Int. J. Pharmaceut. Sci. Rev. Res..

[bib32] Johansson L.H., Borg L.H. (1988). A spectrophotometric method for determination of catalase activity in small tissue samples. Anal. Biochem..

[bib33] Johnston K., Sharp P., Clifford M., Morgan L. (2005). Dietary polyphenols decrease glucose uptake by human intestinal Caco-2 cells. FEBS Lett..

[bib34] Kalra K.L., Grewal H.S., Kahlon S.S. (1989). Bioconversion of kinnow-Mandarin waste into single-cell protein. MIRCEN J. Appl. Microbiol. Biotechnol..

[bib36] Mansour D.F., Abdallah H.M., Ibrahim B.M., Hegazy R.R., Esmail R.S., Abdel-Salam L.O. (2019). The carcinogenic agent diethylnitrosamine induces early oxidative stress, inflammation and proliferation in rat liver, stomach and colon: protective effect of ginger extract. Asian Pac. J. Cancer Prev. APJCP: APJCP..

[bib38] Miranda K.M., Espey M.G., Wink D.A. (2001). A rapid, simple spectrophotometric method for simultaneous detection of nitrate and nitrite. Nitric Oxide.

[bib39] Moharram F.A., Al-Gendy A.A., El-Shenawy S.M., Ibrahim B.M., Zarka M.A. (2018). Phenolic profile, anti-inflammatory, antinociceptive, anti-ulcerogenic and hepatoprotective activities of Pimenta racemosa leaves. BMC Compl. Alternative Med..

[bib40] Mori Y., Koide A., Kobayashi Y., Furukawa F., Hirose M., Nishikawa A. (2003). Effects of cigarette smoke and a heterocyclic amine, MeIQx on cytochrome P-450, mutagenic activation of various carcinogens and glucuronidation in rat liver. Mutagenesis.

[bib42] Mossa A.T., Ibrahim F.M., Mohafrash S.M., Abou Baker D.H., El Gengaihi S. (2015). Protective effect of ethanolic extract of grape pomace against the adverse effects of cypermethrin on weanling female rats. Evid. base Compl. Alternative Med..

[bib43] Mostafa R.E., Ibrahim B.M., Abdel Jaleel G.A. (2016). Neuro-protective effects of Ginkgo biloba leaves extract on cerebral ischemia-reperfusion injury induced experimentally in ovariectomized rats. Int. J. Pharm. Pharmaceut. Sci..

[bib63] Motawi T.K., Hamed M.A., Hashem R.M., Shabana M.H., Ahmed Y.R. (2012). Protective and therapeutic effects of Argyreia speciosa against ethanol-induced gastric ulcer in rats. Zeitschrift für naturforschung c.

[bib59] Parhiz H., Roohbakhsh A., Soltani F., Rezaee R., Iranshahi M. (2015). Antioxidant and anti-inflammatory properties of the citrus flavonoids hesperidin and hesperetin: an updated review of their molecular mechanisms and experimental models. Phytother. Res..

[bib44] Pari Leelavinothan, Karthikeyan Asaithambi, ParamasivamKarthika, Rathinam Ayyasamy (2015). Protective effects of hespe-ridin on oxidative stress, dyslipidaemia and histological changes in iron-induced hepatic and renal toxicity in rats. Toxicol Rep.

[bib47] Reitman S., Frankel S. (1957 Jul 1). A colorimetric method for the determination of serum glutamic oxalacetic and glutamic pyruvic transaminases. Am. J. Clin. Pathol..

[bib60] Roohbakhsh A., Parhiz H., Soltani F., Rezaee R., Iranshahi M. (2015). Molecular mechanisms behind the biological effects of hesperidin and hesperetin for the prevention of cancer and cardiovascular diseases. Life sci..

[bib48] Rouseff R., Perez-Cacho P.R. (2007). Flavours and Fragrances.

[bib49] Ruiz-Larrea M.B., Leal A.M., Liza M., Lacort M., de Groot H. (1994). Antioxidant effects of estradiol and 2-hydroxyestradiol on iron-induced lipid peroxidation of rat liver microsomes. Steroids.

[bib50] Sabiu S., Wudil A.M., Sunmonu T.O. (2014). Combined administration of Telfaira occidentalis and Vernonia amygdalina leaf powders ameliorates garlic-induced hepatotoxicity in Wistar rats. Pharmacologia.

[bib51] Salam M.A., Ibrahim B.M., El-Batran S.E., El-Gengaihi S.E., Baker D.H. (2016). Study of the possible antihypertensive and hypolipidemic effects of an herbal mixture on l-name-induced hypertensive rats. Asian J. Pharmaceut. Clin. Res..

[bib53] Shaaban H., Shafei A.A., Abdel JaleelGehad A., Ibrahim B.M., Hassan A.H. (2016). Effect of a single dose adminstration of wheat bran extract and its active components on acute is chemic brain injury. Int. J. Phan-nacognosy Phytochem. Res..

[bib54] Singleton V.L., Rossi J.A. (1965). Colorimetry of total phenolics with phosphomolybdic-phosphotungstic acid reagents. Am. J. Enol. Vitic..

[bib57] Sorg D.A., Buckner B. (1964). A simple method of obtaining venous blood from small laboratory animals. Proceed. Soc. Exp. Biol. Med..

[bib61] Sowmya N., Lakshmipriya N., Arumugam K., Venkatachalam S., Vijayalakshmi P., Ruchi V., Geetha G., Anjana R.M., Mohan V., Krishnaswamy K., Sudha V. (2016). Comparison of dietary profile of a rural south Indian population with the current dietary recommendations for prevention of non-communicable diseases (CURES 147). Indian J. Med. Res..

[bib55] Taha K.F., Hetta M.H., Bakeer W.I., Yassin N.A.Z., Ibrahim B.M.M., Hassan M.E.S. (2016). Comparative phytochemical and pharmacological study of antitussive and antimicrobial effects of Boswellia and thyme essential oils. Der PhannaChemica.

[bib62] Tyśkiewicz K., Konkol M., Kowalski R., Rój E., Warmiński K., Krzyżaniak M., Gil Ł., Stolarski M.J. (2019). Characterization of bioactive compounds in the biomass of black locust, poplar and willow. Trees.

