# Corrigendum to “Biochemical and pharmacological prospects of citrus sinensis peel” [Heliyon 8, (8), (August 2022), e09979]

**DOI:** 10.1016/j.heliyon.2022.e12060

**Published:** 2022-12-05

**Authors:** Doha H. Abou Baker, Bassant M.M. Ibrahim, Yasmin Abdel-Latif, Nabila S. Hassan, Emad M. Hassan, Souad El Gengaihi

**Affiliations:** a Medicinal and Aromatic Plants Department, Pharmaceutical and Drug Industries Institute, National Research Centre, Dokki, Giza, PO 12622, Egypt; b Pharmacology Department, Medicine and Clinical Studies Research Institute, National Research Centre, Dokki, Giza, PO 12622, Egypt; c Medical biochemistry Department, National Research Centre, Cairo, PO 12622, Egypt; d Faculty of Biotechnology, October University for Modern Sciences and Arts, 6th October, Giza, Egypt; e Pathology Department, Medical Research Institute, National Research Centre, Dokki, Giza, PO 12622, Egypt

In the original published version of this article, co-author Yasmin Abdel-Latif affiliations are incorrect. The correct affiliations can be found below. The authors apologize for the errors. Both the HTML and PDF versions of the article have been updated to correct the errors.

Yasmin Abdel-Latif ^c,d^
^c^ Medical Biochemistry Department, Medical Research Institute, National Research Centre, Dokki, Giza, PO 12622, Egypt
^d^ Faculty of Biotechnology, October University for Modern Sciences and Arts, 6th October, Giza, Egypt
Nabila S. Hassan^e^
^e^ Pathology Department, Medical Research Institute, National Research Centre, Dokki, Giza, PO 12622, Egypt

## Declaration of interest’s statement

The authors declare no conflict of interest.

